# Supplementary figures and images for: miR-344d-3p regulates osteogenic and adipogenic differentiation of mouse mandibular bone marrow mesenchymal stem cells
Source: PeerJ. 2023 Feb 14;11:e14838. doi: 10.7717/peerj.14838 (PMC9936866; doi:10.7717/peerj.14838)

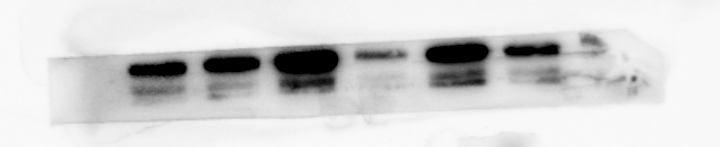

Supplement: Supplemental Information 2 — From left to right in lane 3 and 4 [file peerj-11-14838-s002.png]

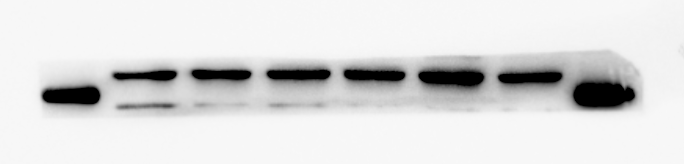

Supplement: Supplemental Information 3 — From left to right in lane 3 and 4 [file peerj-11-14838-s003.png]

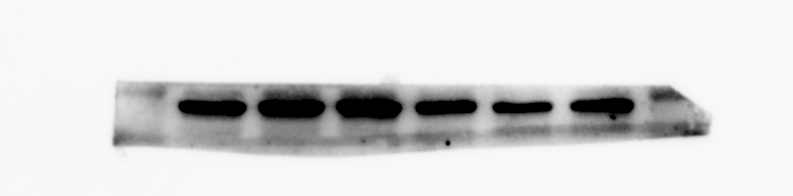

Supplement: Supplemental Information 4 — From left to right in lane 5 and 6 [file peerj-11-14838-s004.png]

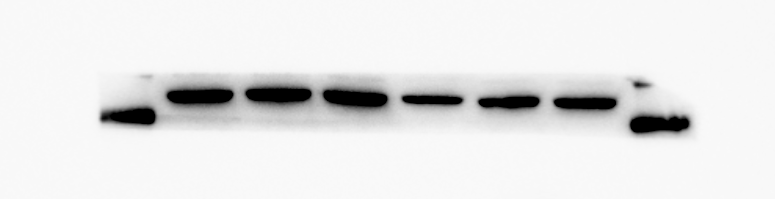

Supplement: Supplemental Information 5 — From left to right in lane 5 and 6 [file peerj-11-14838-s005.png]

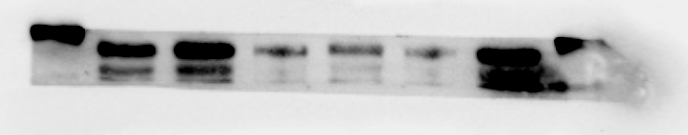

Supplement: Supplemental Information 6 — From left to right in lane 1 to 3 [file peerj-11-14838-s006.png]

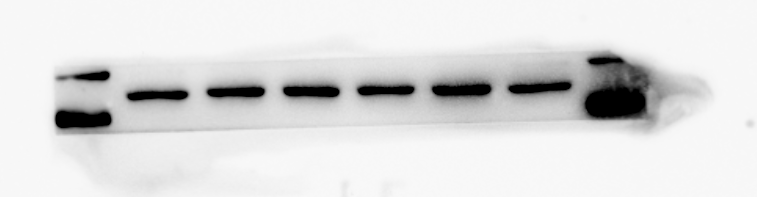

Supplement: Supplemental Information 7 — From left to right in lane 1 to 3 [file peerj-11-14838-s007.png]

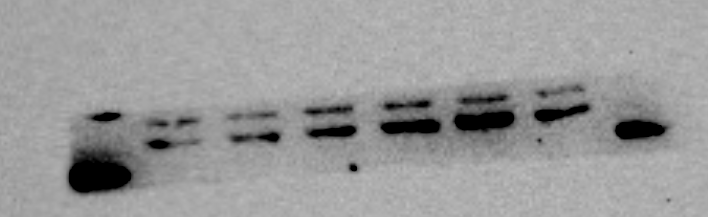

Supplement: Supplemental Information 8 — From left to right in lane 4 to 6 [file peerj-11-14838-s008.png]

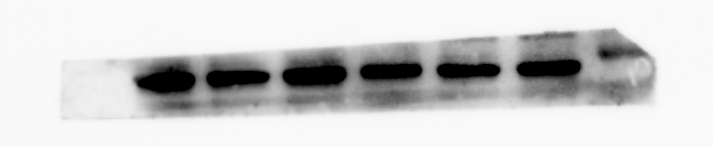

Supplement: Supplemental Information 9 — From left to right in lane 4 to 6 [file peerj-11-14838-s009.png]

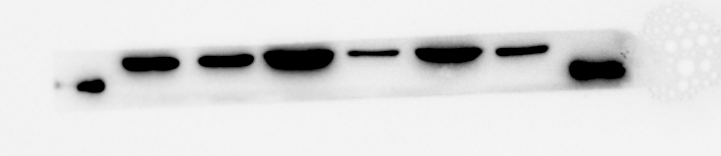

Supplement: Supplemental Information 10 — From left to right in lane 1 to 3 [file peerj-11-14838-s010.png]

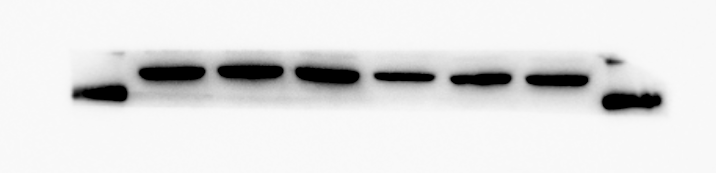

Supplement: Supplemental Information 11 — From left to right in lane 1 to 3 [file peerj-11-14838-s011.png]

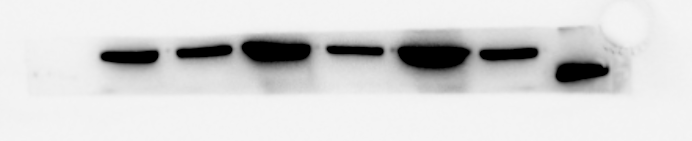

Supplement: Supplemental Information 12 — From left to right in lane 1 to 3 [file peerj-11-14838-s012.png]

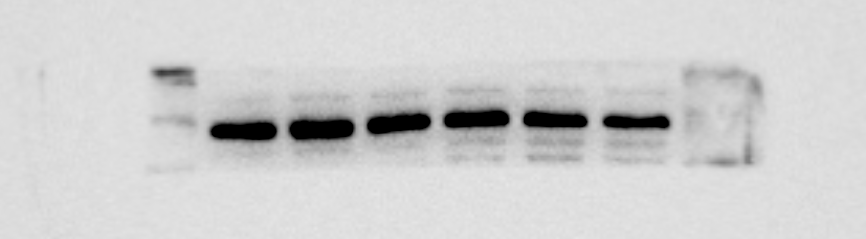

Supplement: Supplemental Information 13 — From left to right in lane 1 to 3 [file peerj-11-14838-s013.png]

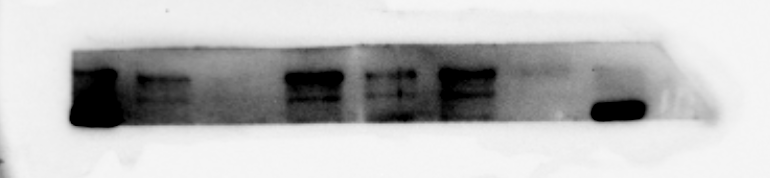

Supplement: Supplemental Information 14 — From left to right in lane 1 to 3 [file peerj-11-14838-s014.png]

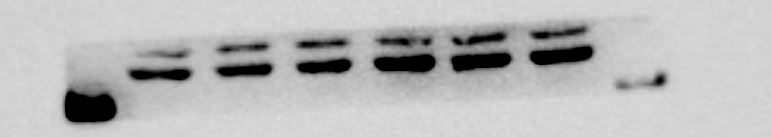

Supplement: Supplemental Information 15 — From left to right in lane 1 to 3 [file peerj-11-14838-s015.png]

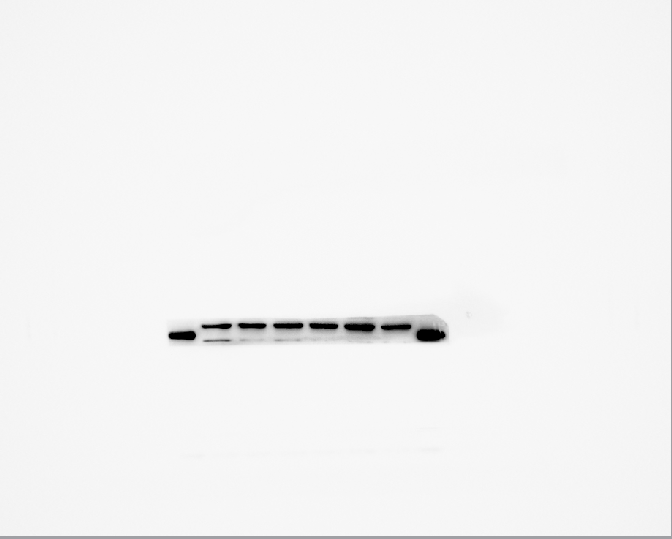

Supplement: Figure S1 — Raw data: Figure 1 A-H [file peerj-11-14838-s018.zip › Figure 2/D/a┬-actin,line3,4]

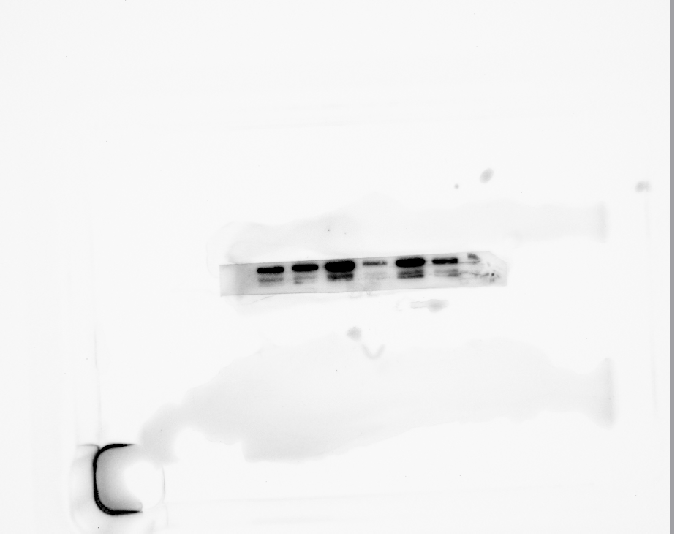

Supplement: Figure S1 — Raw data: Figure 1 A-H [file peerj-11-14838-s018.zip › Figure 2/D/RUNX2ú1⁄4line3,4]

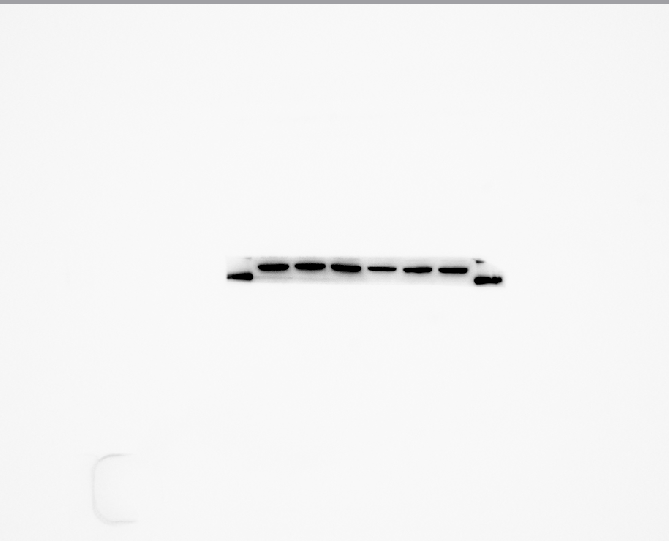

Supplement: Figure S1 — Raw data: Figure 1 A-H [file peerj-11-14838-s018.zip › Figure 2/H/a┬-actin,line5,6]

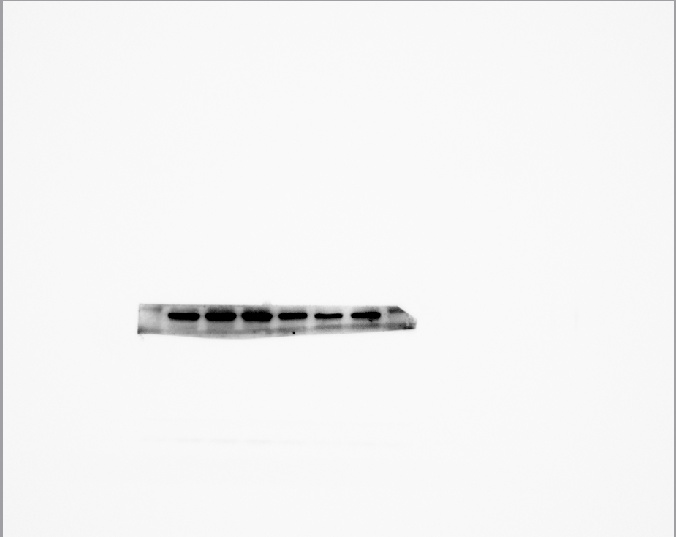

Supplement: Figure S1 — Raw data: Figure 1 A-H [file peerj-11-14838-s018.zip › Figure 2/H/PPAR-a├ú1⁄4line5,6]

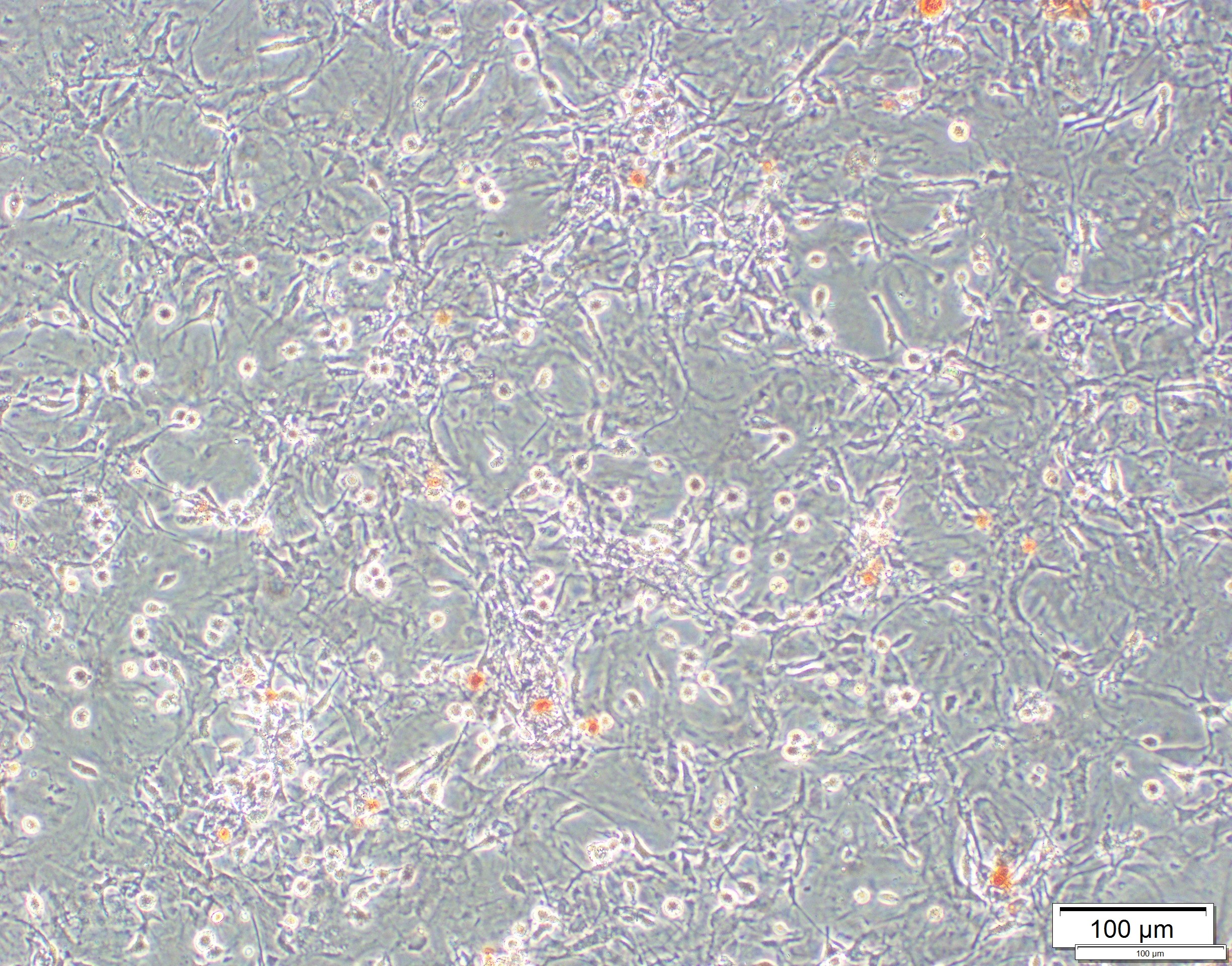

Supplement: Figure S1 — Raw data: Figure 1 A-H [file peerj-11-14838-s018.zip › Figure 2/A/OVX/1.jpg]

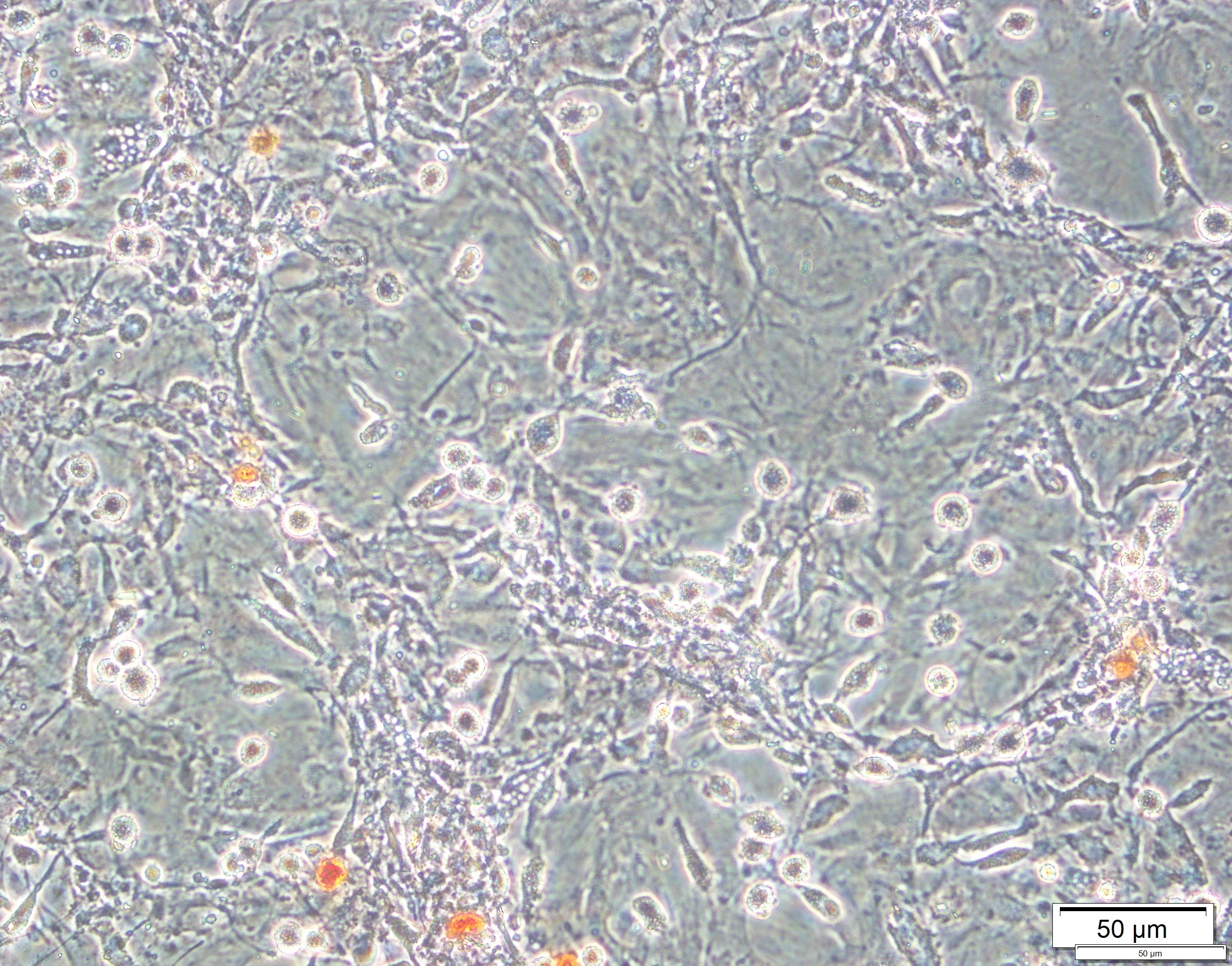

Supplement: Figure S1 — Raw data: Figure 1 A-H [file peerj-11-14838-s018.zip › Figure 2/A/OVX/2.jpg]

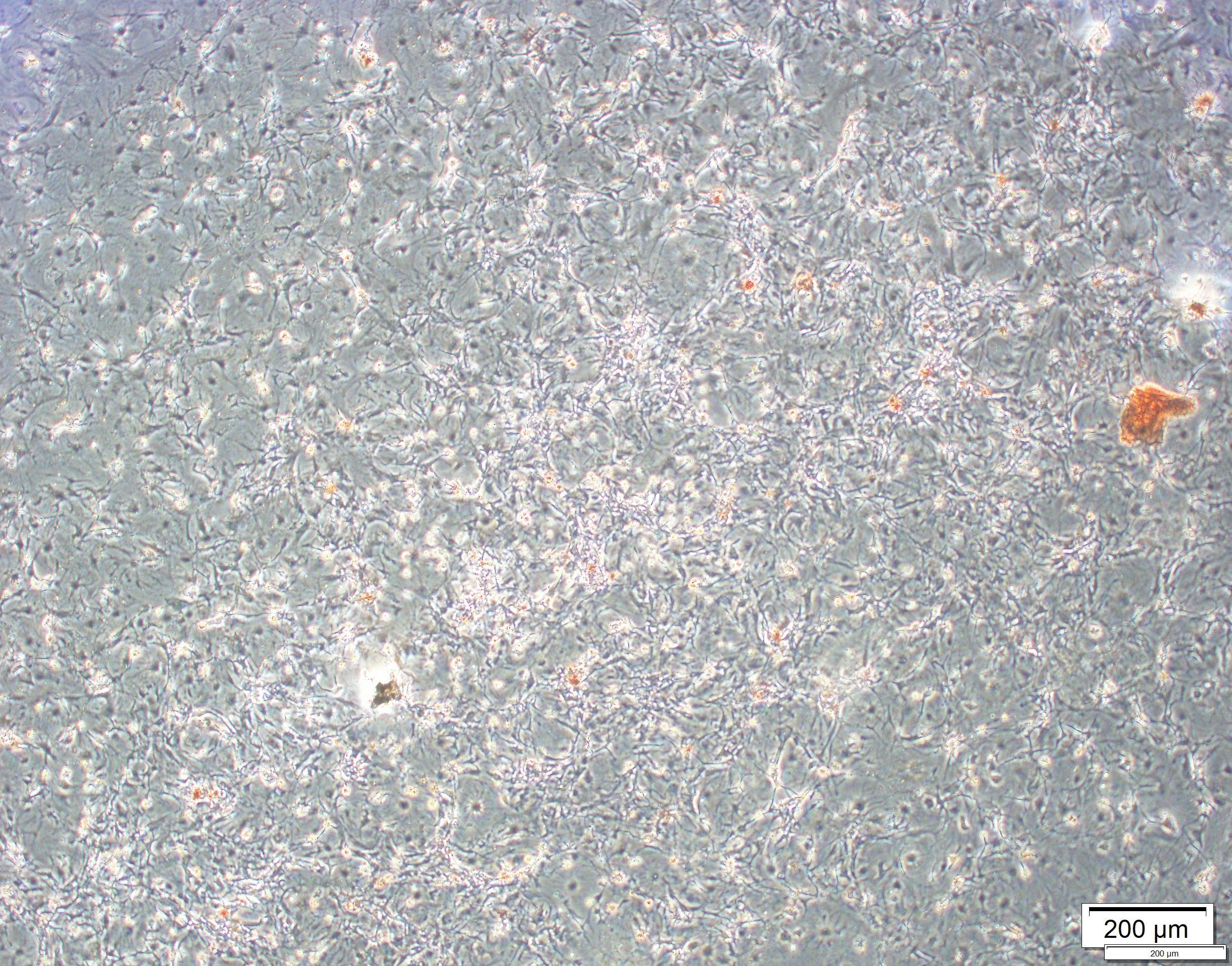

Supplement: Figure S1 — Raw data: Figure 1 A-H [file peerj-11-14838-s018.zip › Figure 2/A/OVX/3.jpg]

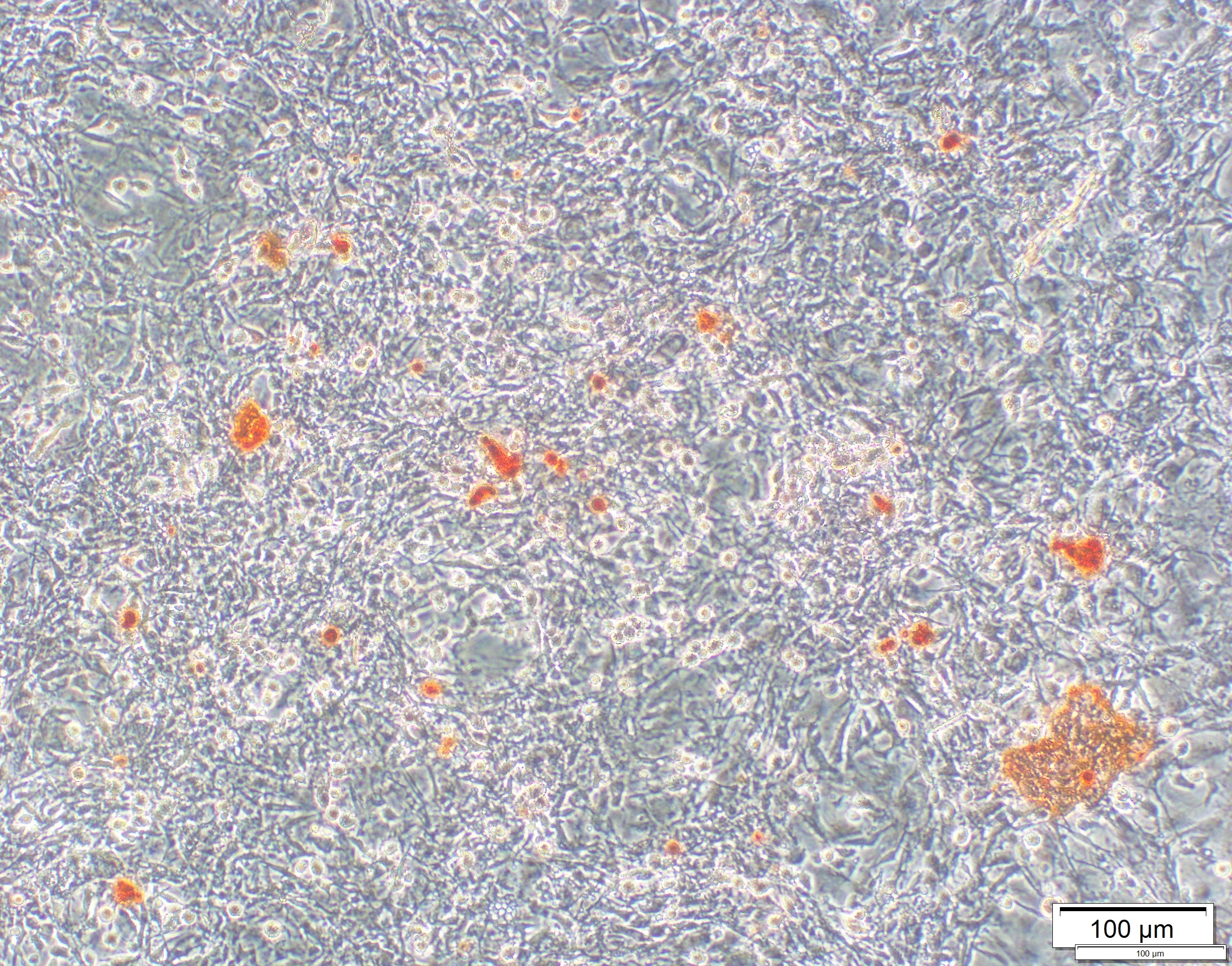

Supplement: Figure S1 — Raw data: Figure 1 A-H [file peerj-11-14838-s018.zip › Figure 2/A/SHAM/1.jpg]

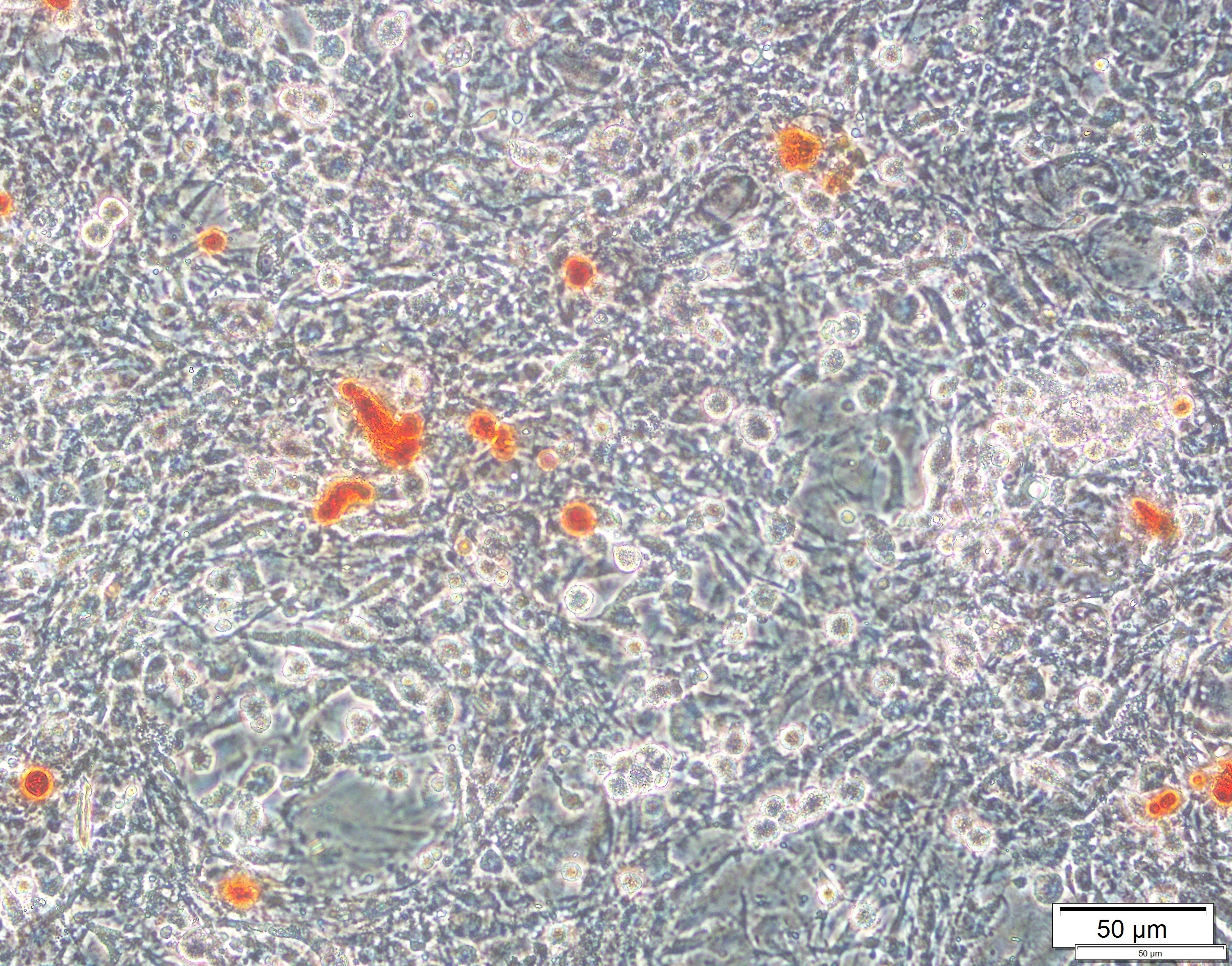

Supplement: Figure S1 — Raw data: Figure 1 A-H [file peerj-11-14838-s018.zip › Figure 2/A/SHAM/2.jpg]

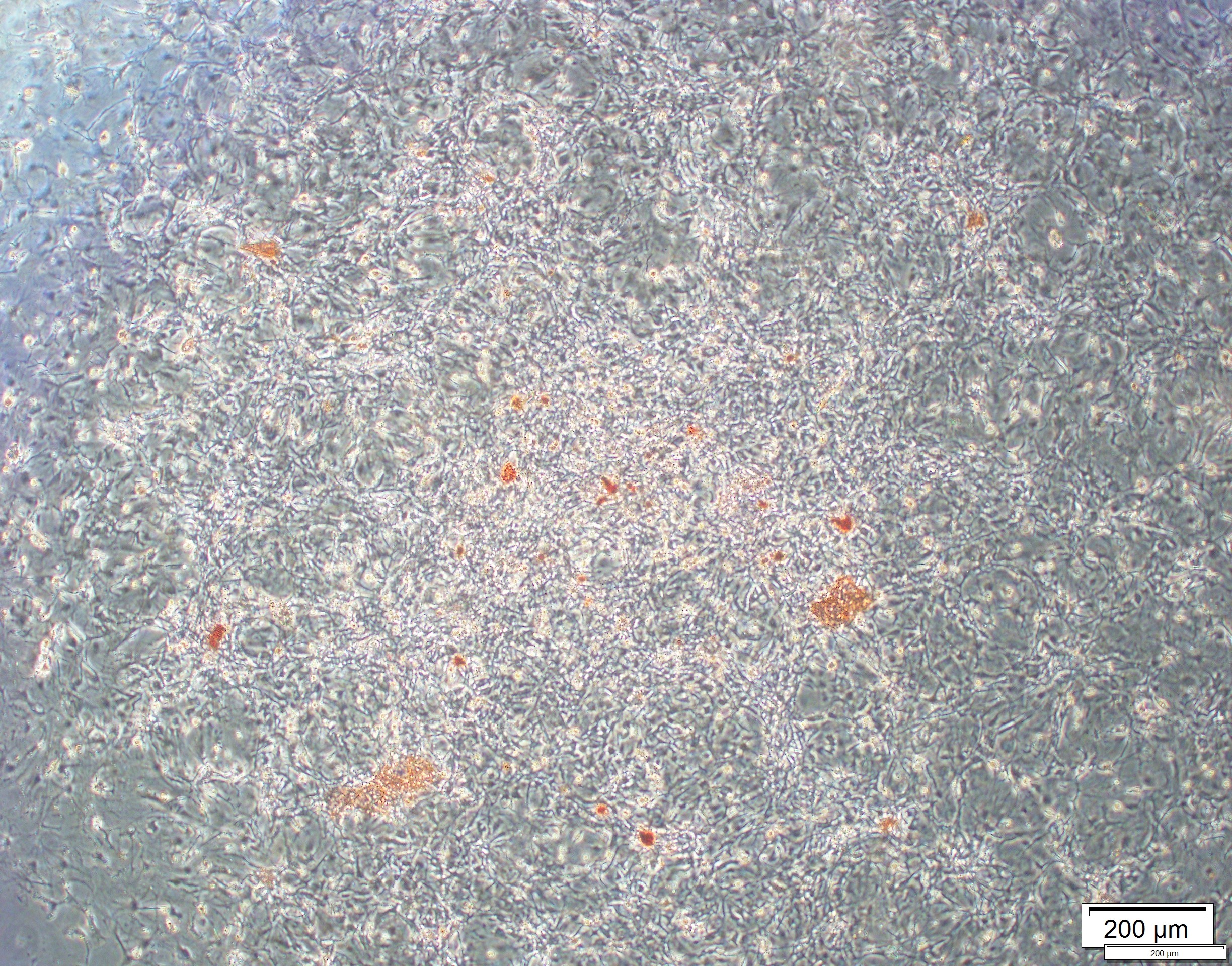

Supplement: Figure S1 — Raw data: Figure 1 A-H [file peerj-11-14838-s018.zip › Figure 2/A/SHAM/3.jpg]

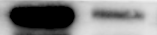

Supplement: Figure S1 — Raw data: Figure 1 A-H [file peerj-11-14838-s018.zip › Figure 2/D/screenshots/RUNX2.png]

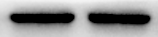

Supplement: Figure S1 — Raw data: Figure 1 A-H [file peerj-11-14838-s018.zip › Figure 2/D/screenshots/β-actin.png]

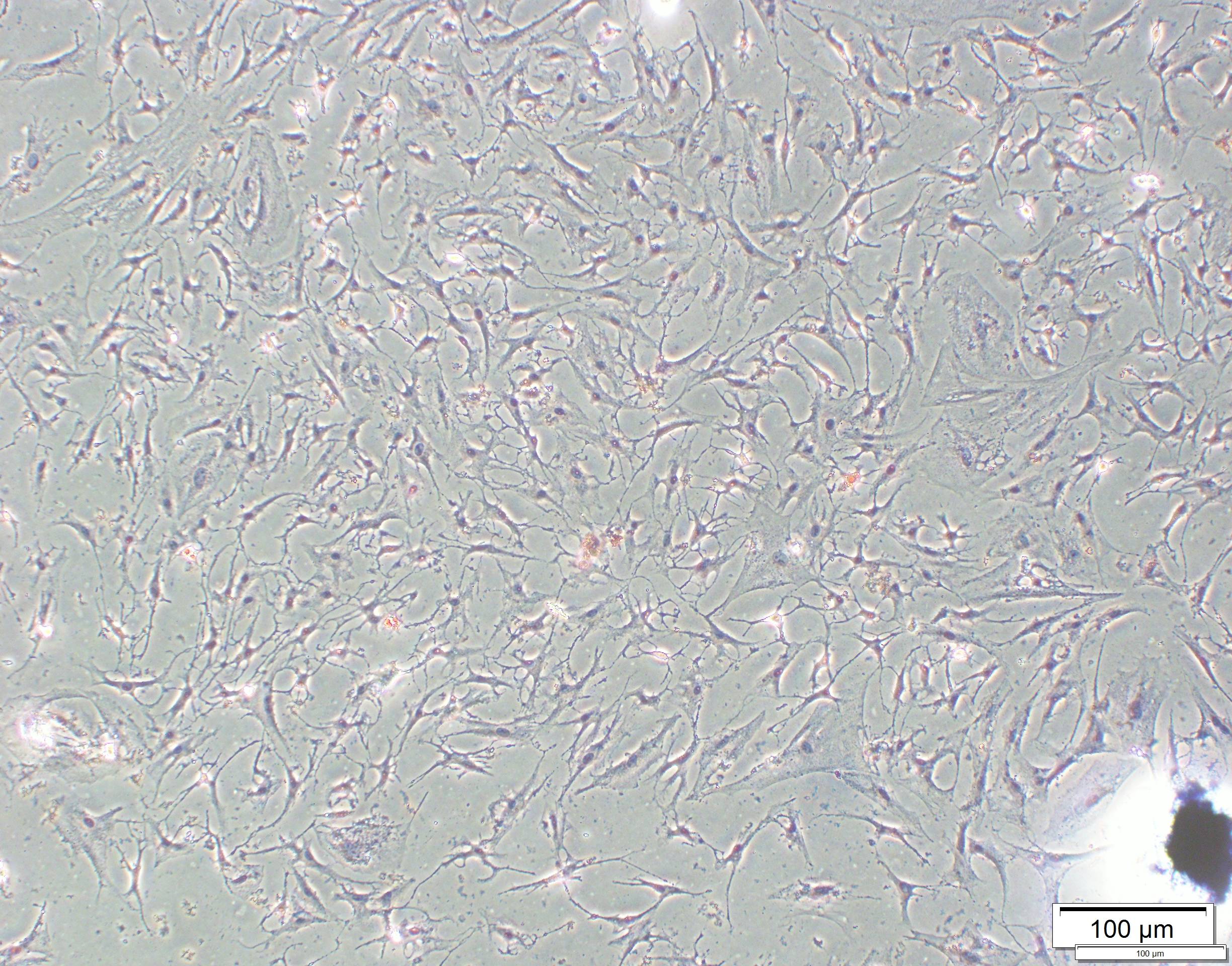

Supplement: Figure S1 — Raw data: Figure 1 A-H [file peerj-11-14838-s018.zip › Figure 2/E/OVX/1.jpg]

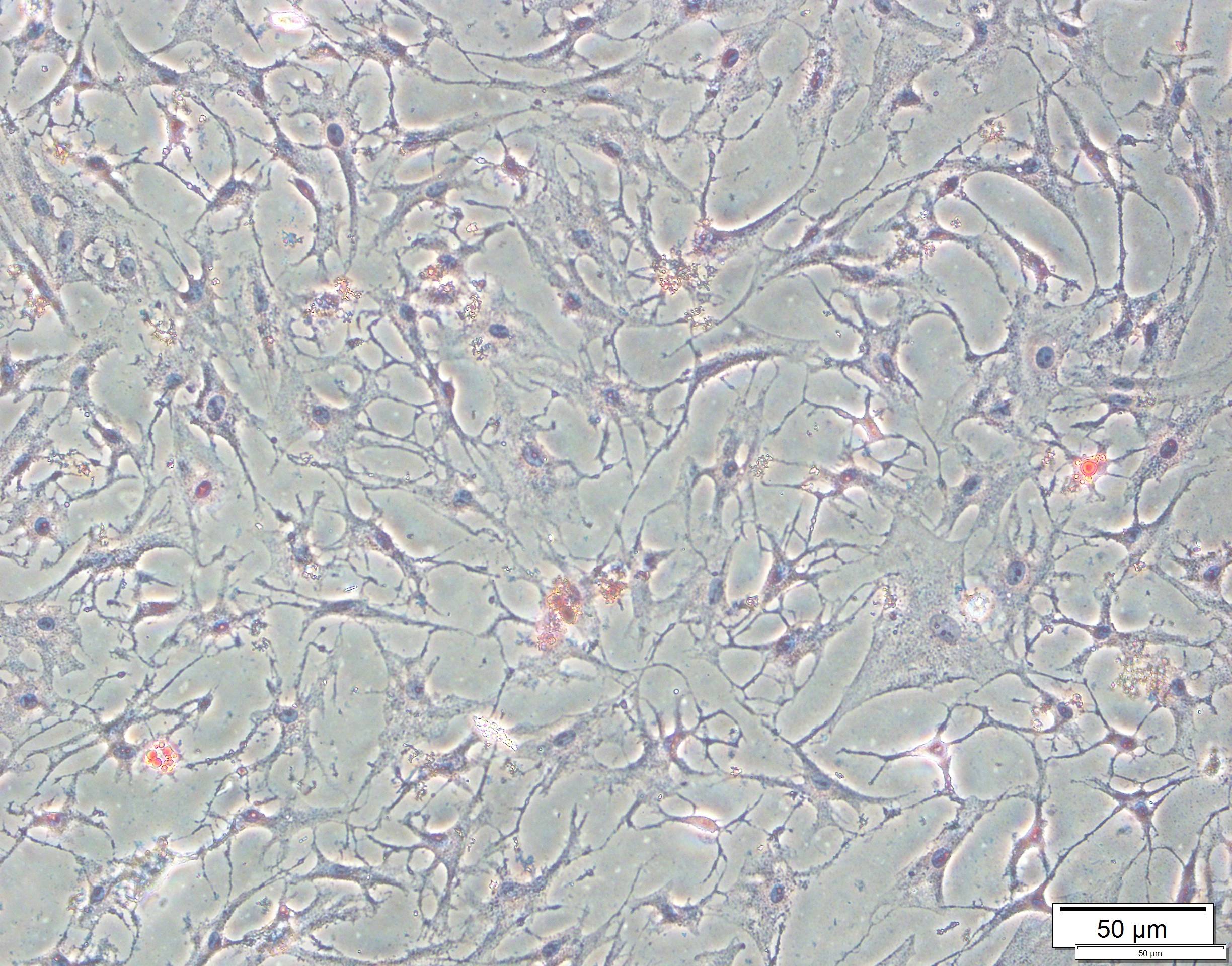

Supplement: Figure S1 — Raw data: Figure 1 A-H [file peerj-11-14838-s018.zip › Figure 2/E/OVX/图像_32690.jpg]

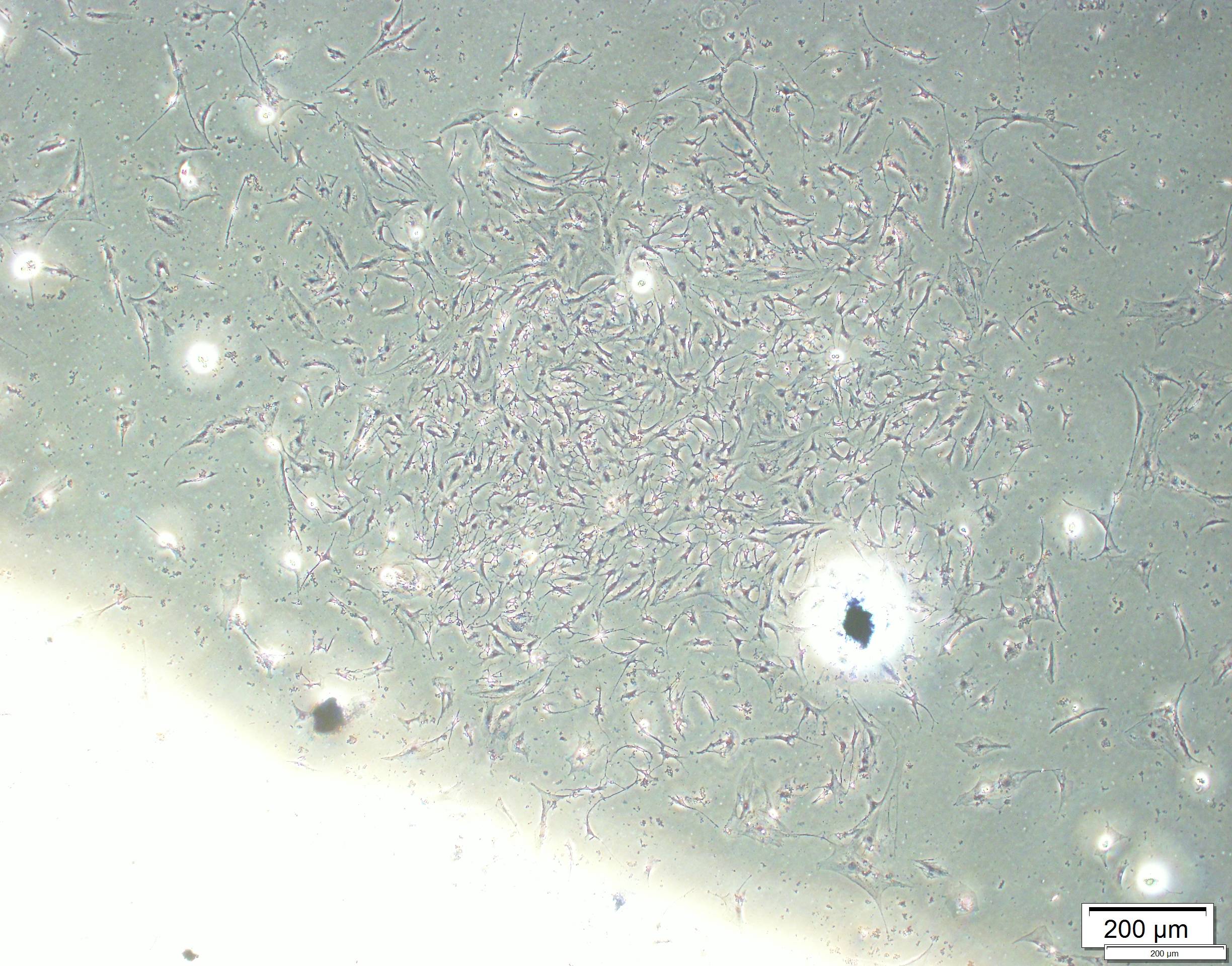

Supplement: Figure S1 — Raw data: Figure 1 A-H [file peerj-11-14838-s018.zip › Figure 2/E/OVX/图像_32691.jpg]

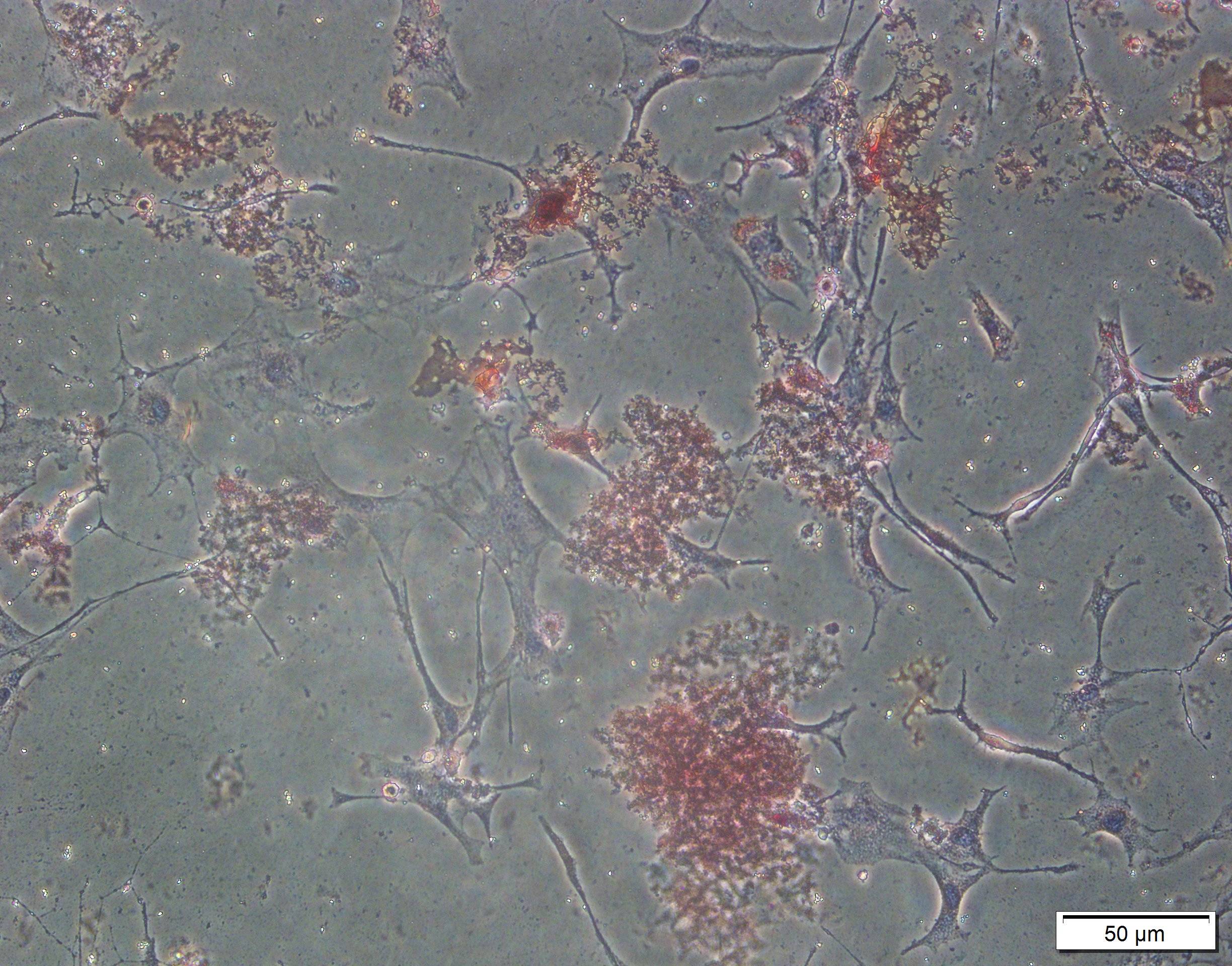

Supplement: Figure S1 — Raw data: Figure 1 A-H [file peerj-11-14838-s018.zip › Figure 2/E/OVX/图像_35785.jpg]

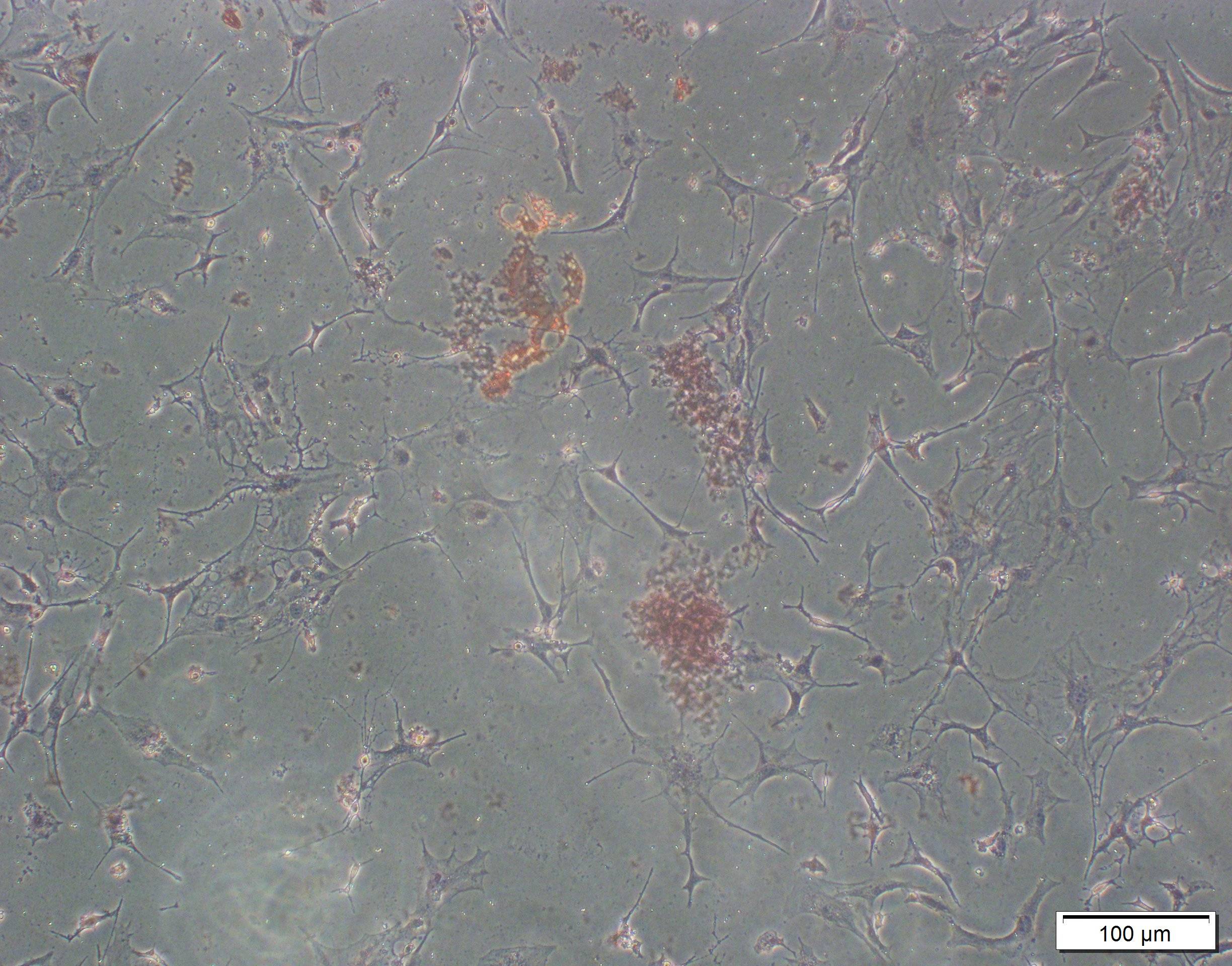

Supplement: Figure S1 — Raw data: Figure 1 A-H [file peerj-11-14838-s018.zip › Figure 2/E/OVX/图像_35786.jpg]

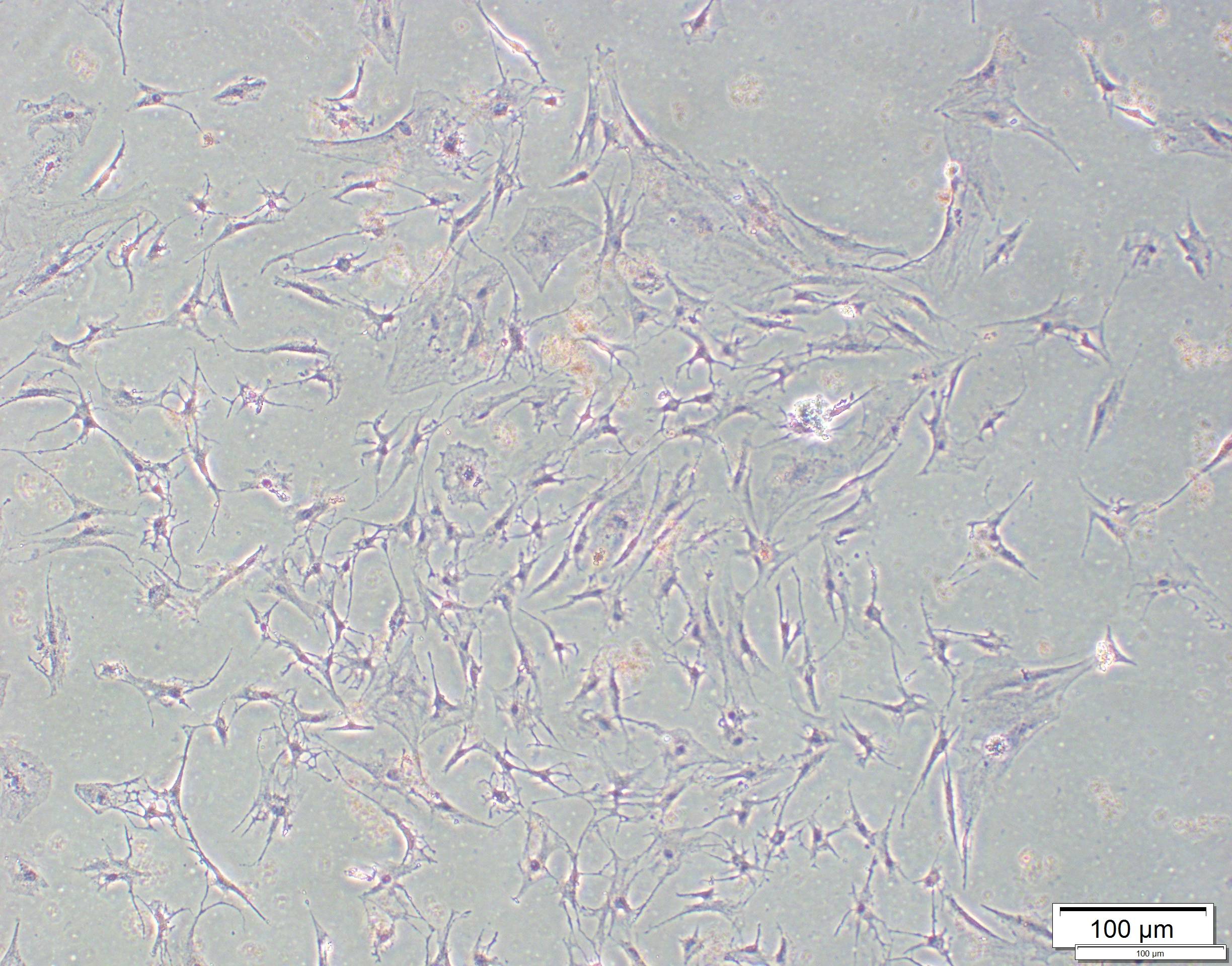

Supplement: Figure S1 — Raw data: Figure 1 A-H [file peerj-11-14838-s018.zip › Figure 2/E/SHAM/图像_32698.jpg]

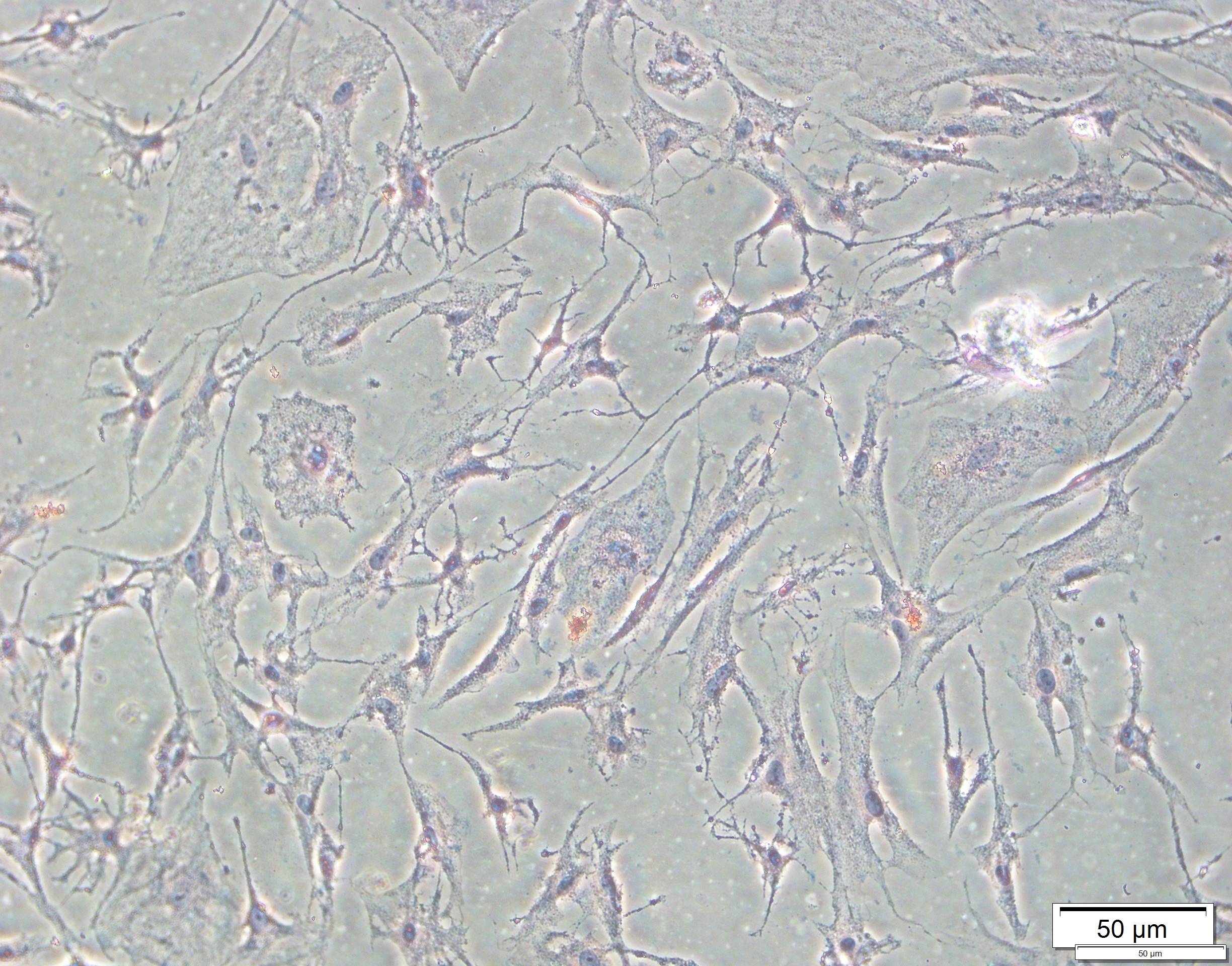

Supplement: Figure S1 — Raw data: Figure 1 A-H [file peerj-11-14838-s018.zip › Figure 2/E/SHAM/图像_32699.jpg]

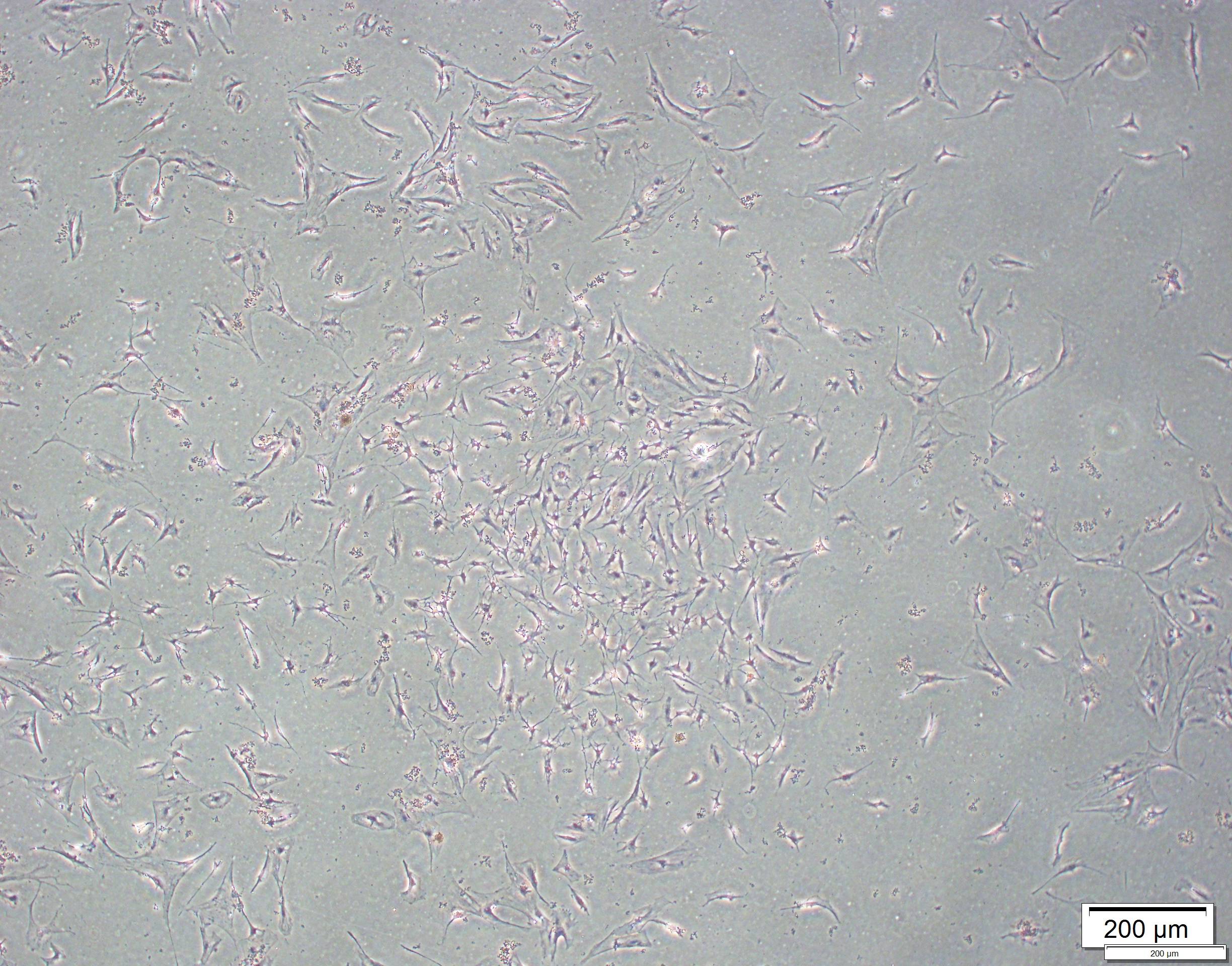

Supplement: Figure S1 — Raw data: Figure 1 A-H [file peerj-11-14838-s018.zip › Figure 2/E/SHAM/图像_32700.jpg]

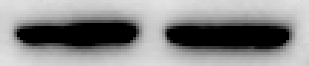

Supplement: Figure S1 — Raw data: Figure 1 A-H [file peerj-11-14838-s018.zip › Figure 2/H/screenshots/actin.png]

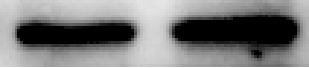

Supplement: Figure S1 — Raw data: Figure 1 A-H [file peerj-11-14838-s018.zip › Figure 2/H/screenshots/PPAR.png]

Color Key

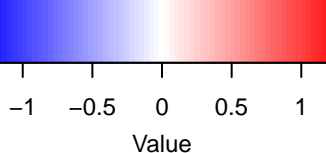

OhevsShe p<0.01

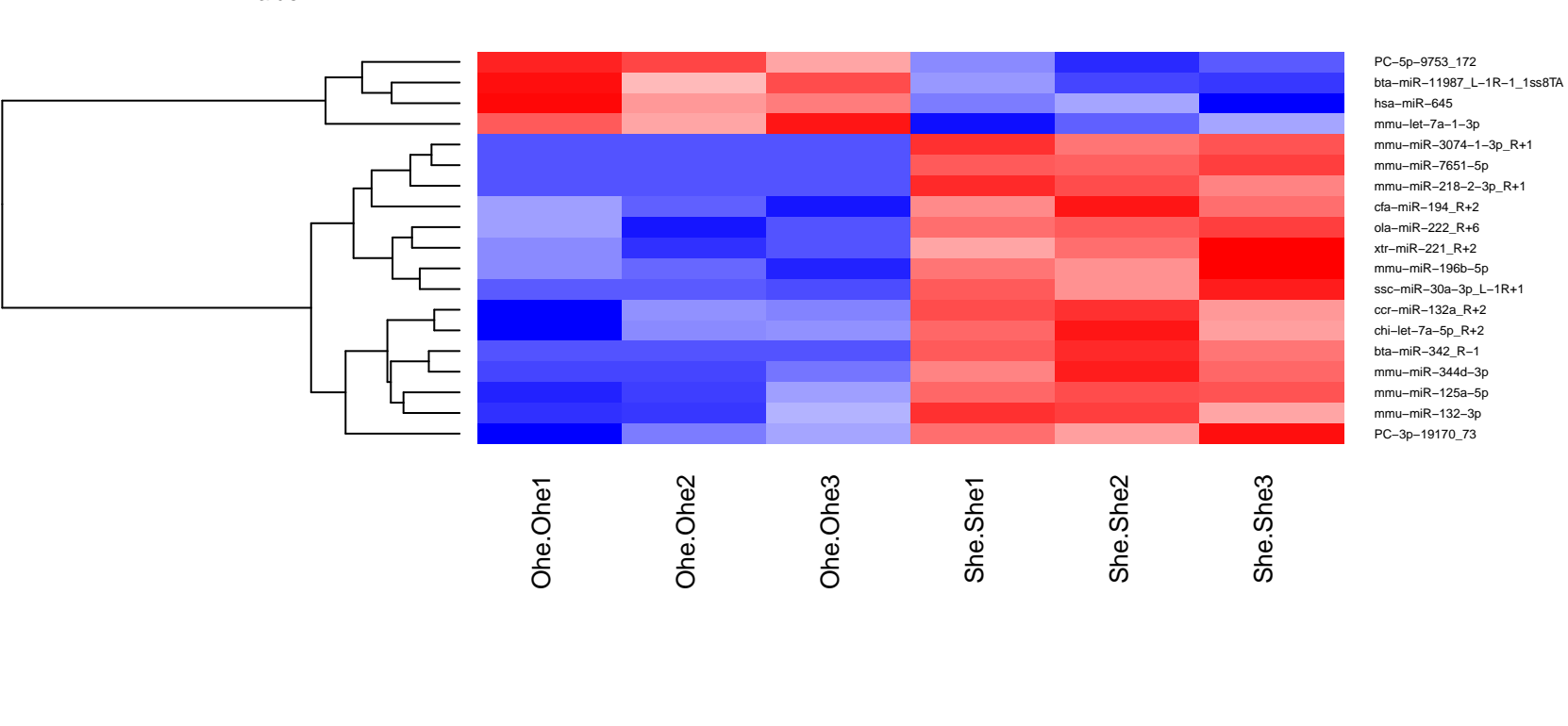

Color Key

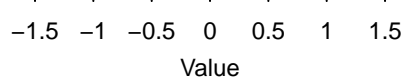

OhevsShe p<0.05

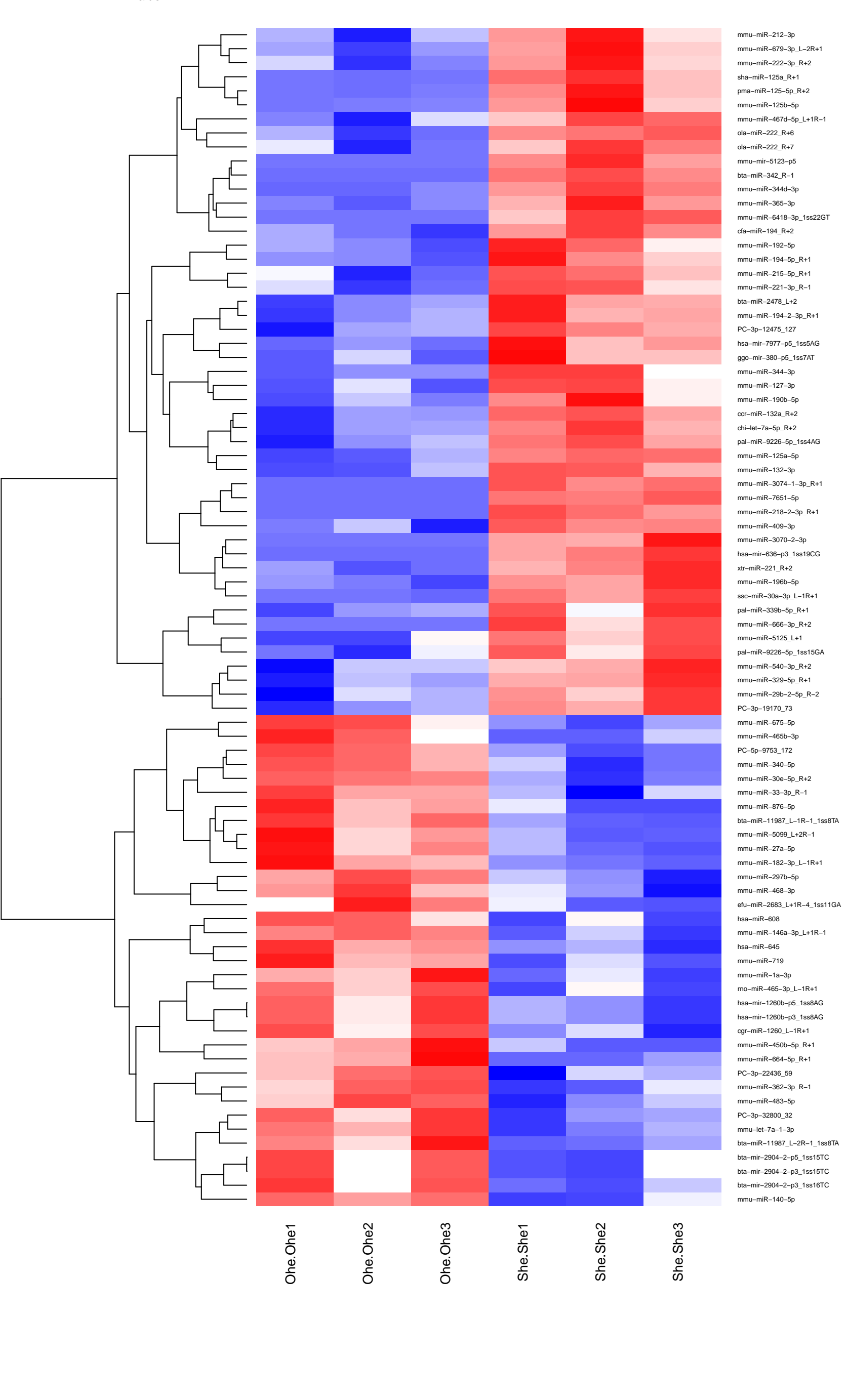

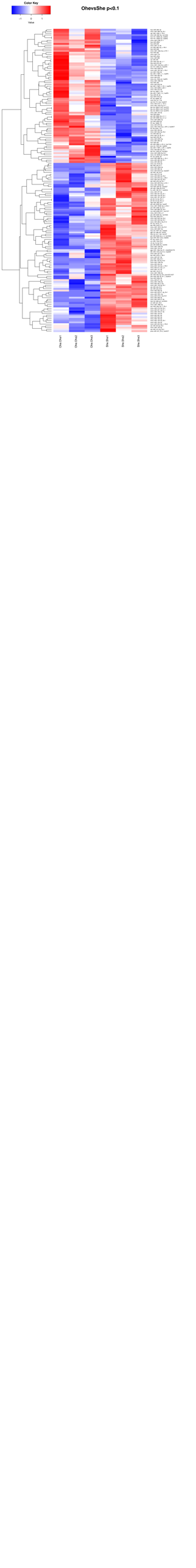

Supplement: Figure S2 — Raw data: Figure 2 A-D [file peerj-11-14838-s019.zip › Figure 3/A/heatmap_of_differentially_expressed_miRNAs(original data).pdf]

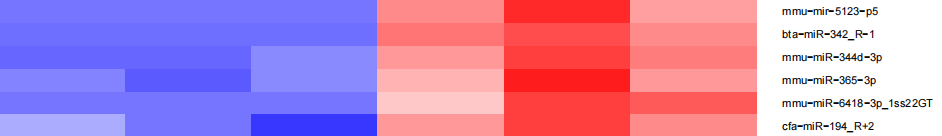

Supplement: Figure S2 — Raw data: Figure 2 A-D [file peerj-11-14838-s019.zip › Figure 3/A/screenshots.jpg]

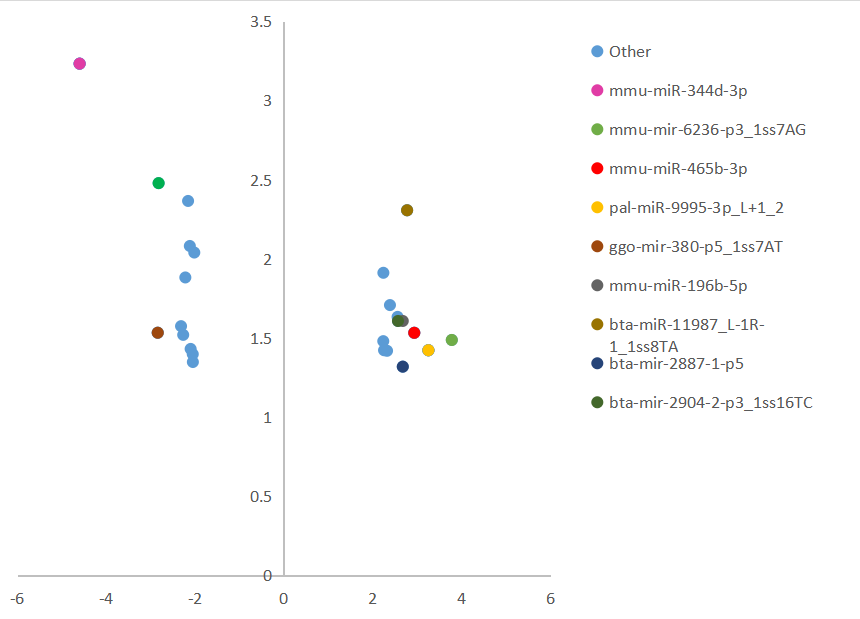

Supplement: Figure S2 — Raw data: Figure 2 A-D [file peerj-11-14838-s019.zip › Figure 3/B/screenshots.jpg]

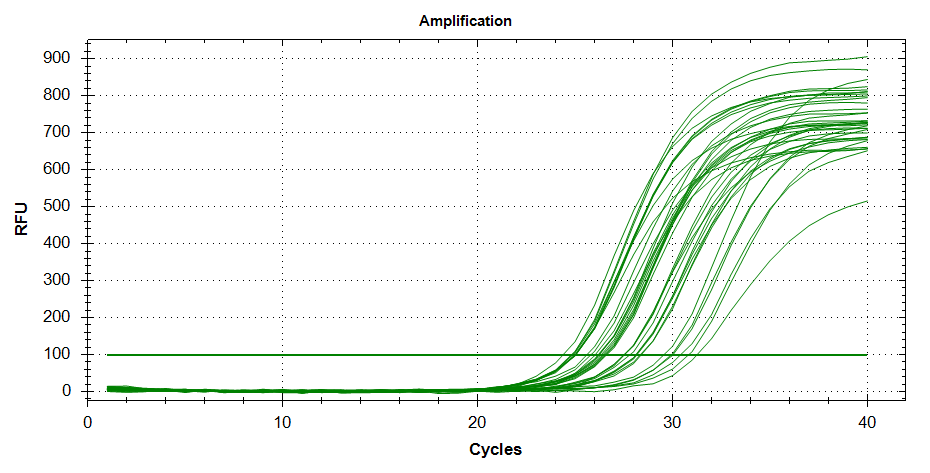

Supplement: Figure S2 — Raw data: Figure 2 A-D [file peerj-11-14838-s019.zip › Figure 3/D/original data/Amplification curve/MIR-344D-3P.png]

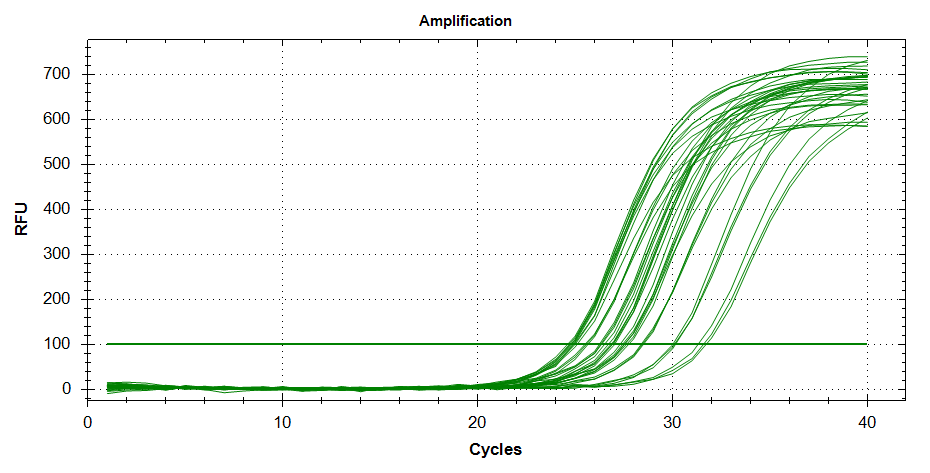

Supplement: Figure S2 — Raw data: Figure 2 A-D [file peerj-11-14838-s019.zip › Figure 3/D/original data/Amplification curve/MIR-465-3P.png]

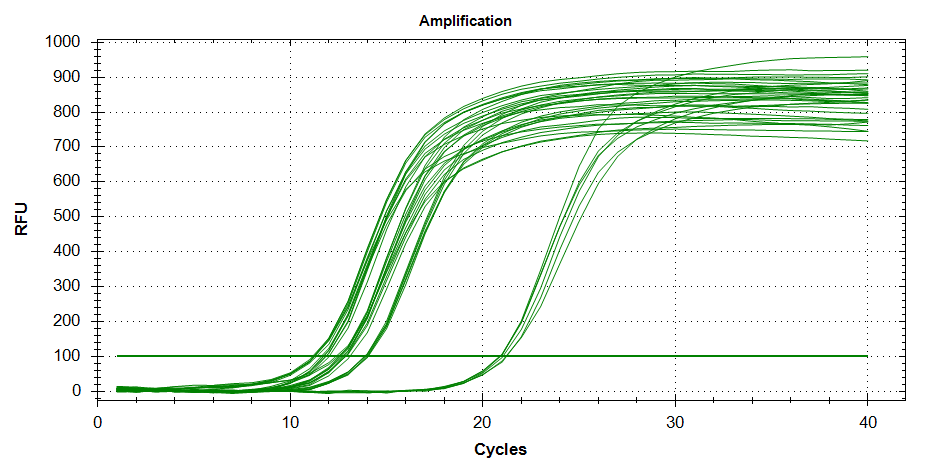

Supplement: Figure S2 — Raw data: Figure 2 A-D [file peerj-11-14838-s019.zip › Figure 3/D/original data/Amplification curve/U6.png]

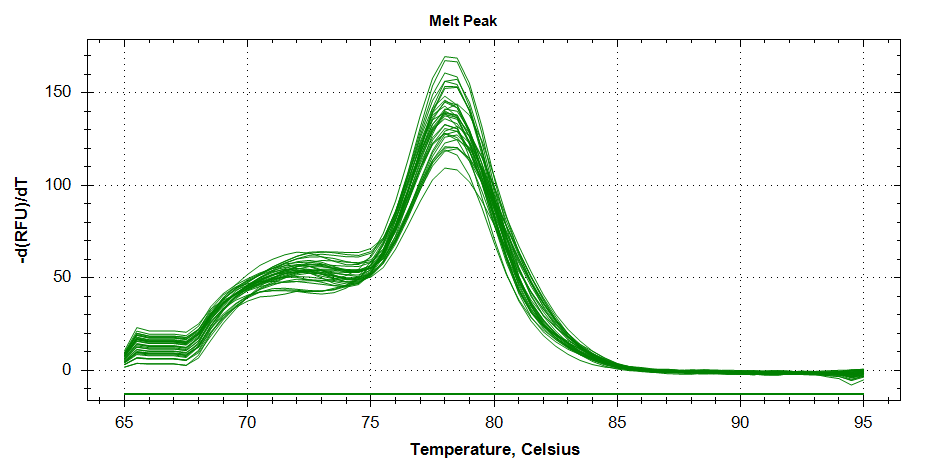

Supplement: Figure S2 — Raw data: Figure 2 A-D [file peerj-11-14838-s019.zip › Figure 3/D/original data/Dissolve curve/MIR-344D-3P.png]

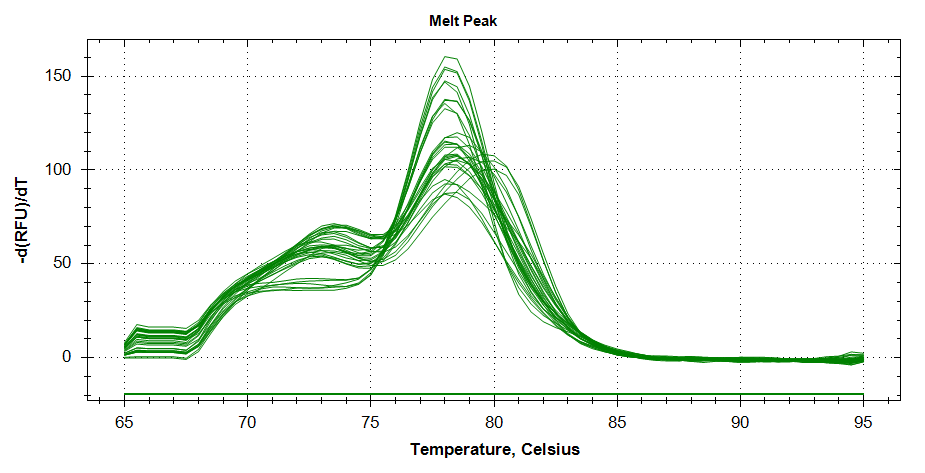

Supplement: Figure S2 — Raw data: Figure 2 A-D [file peerj-11-14838-s019.zip › Figure 3/D/original data/Dissolve curve/MIR-465-3P.png]

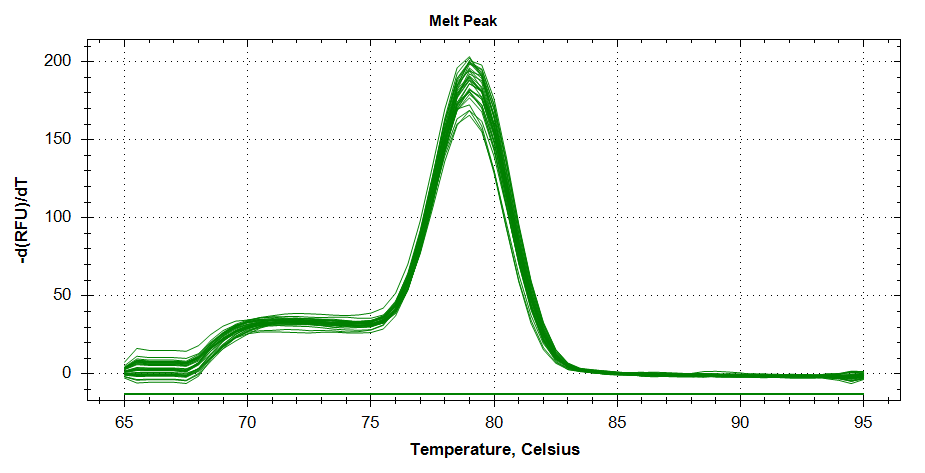

Supplement: Figure S2 — Raw data: Figure 2 A-D [file peerj-11-14838-s019.zip › Figure 3/D/original data/Dissolve curve/U6.png]

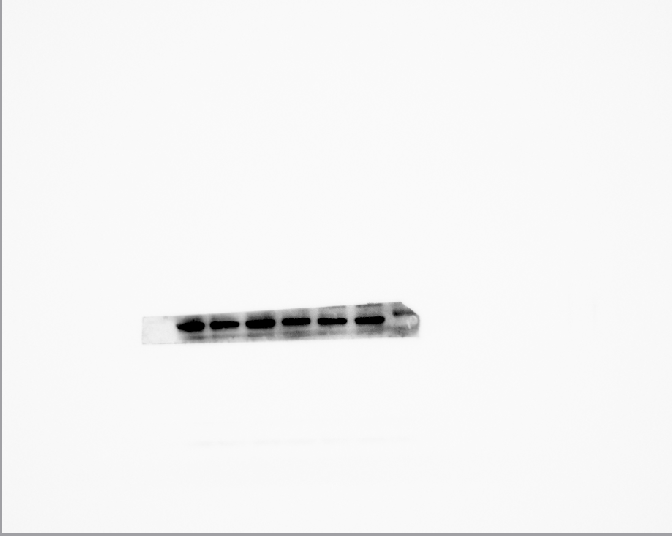

Supplement: Figure S3 — Raw data: Figure 3 A-F [file peerj-11-14838-s020.zip › Figure 4/F/a┬-actin,line4-6]

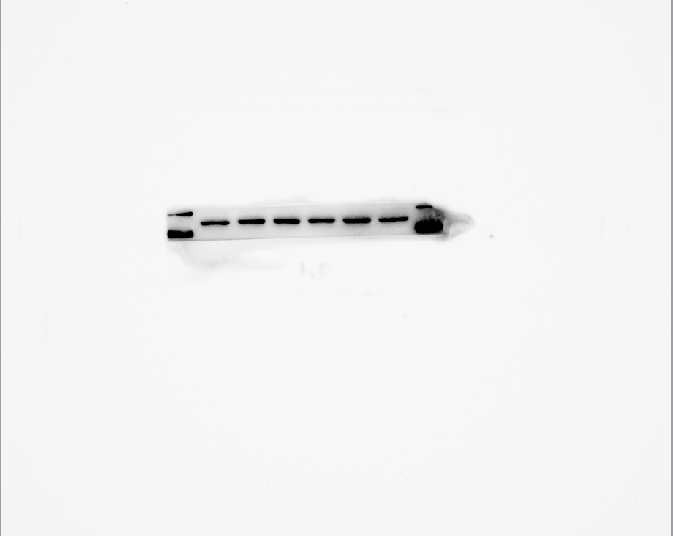

Supplement: Figure S3 — Raw data: Figure 3 A-F [file peerj-11-14838-s020.zip › Figure 4/E/a┬-actin,line1-3]

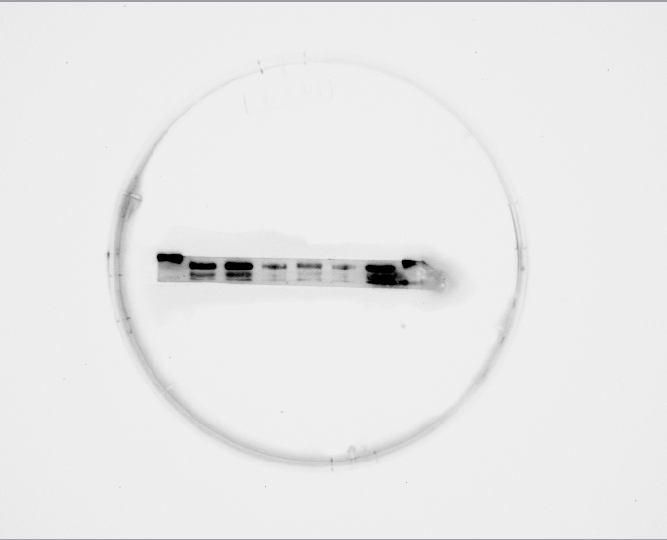

Supplement: Figure S3 — Raw data: Figure 3 A-F [file peerj-11-14838-s020.zip › Figure 4/E/runx2,line1-3]

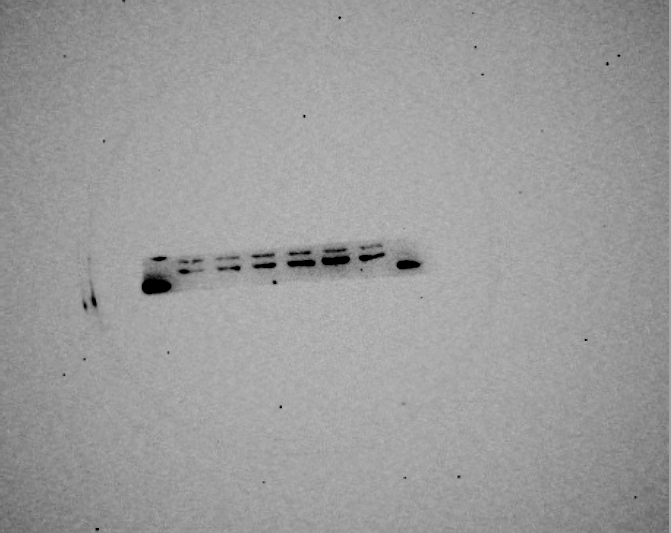

Supplement: Figure S3 — Raw data: Figure 3 A-F [file peerj-11-14838-s020.zip › Figure 4/F/runx2,line4-6]

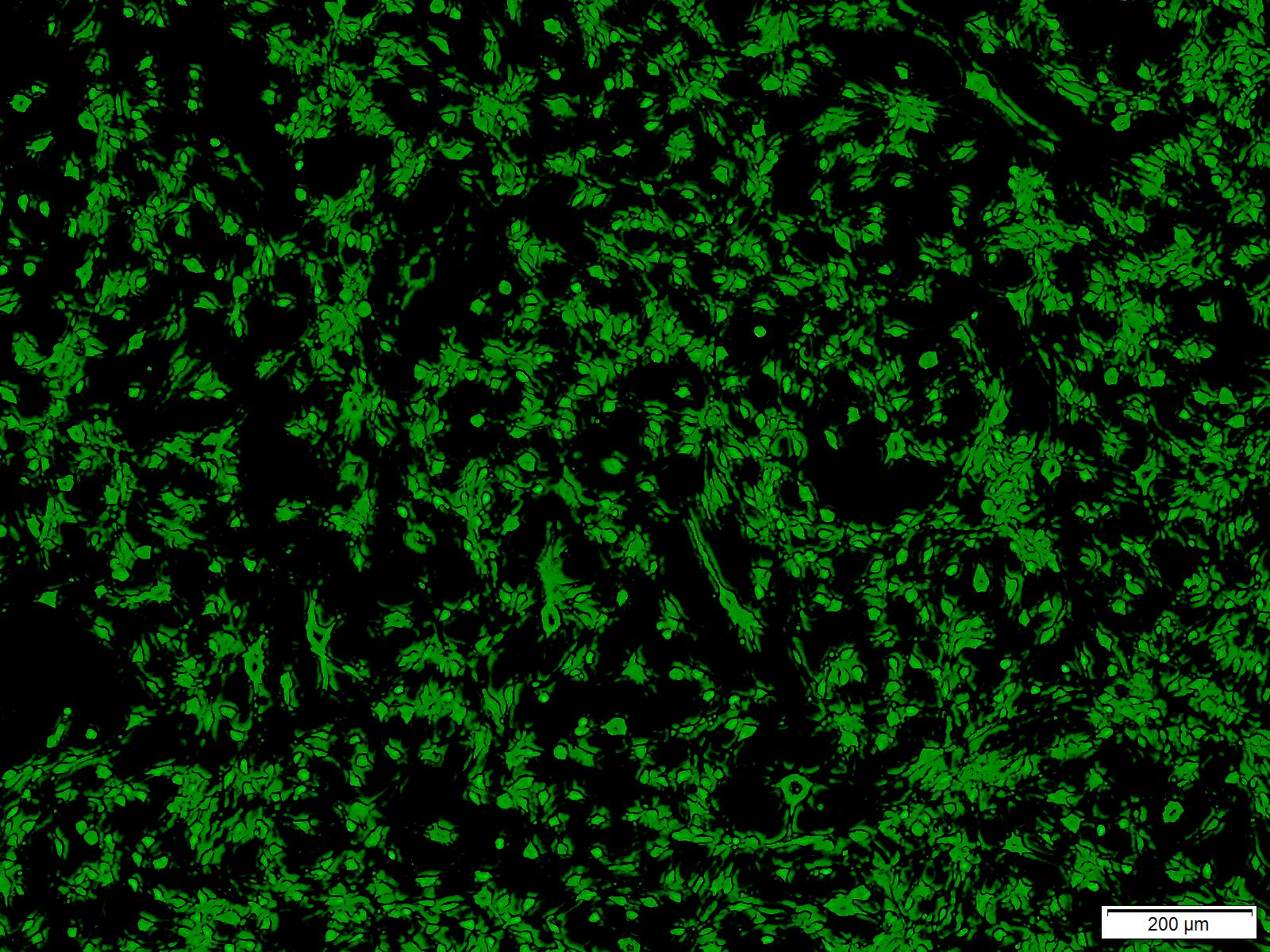

Supplement: Figure S3 — Raw data: Figure 3 A-F [file peerj-11-14838-s020.zip › Figure 4/A/control(1).jpg]

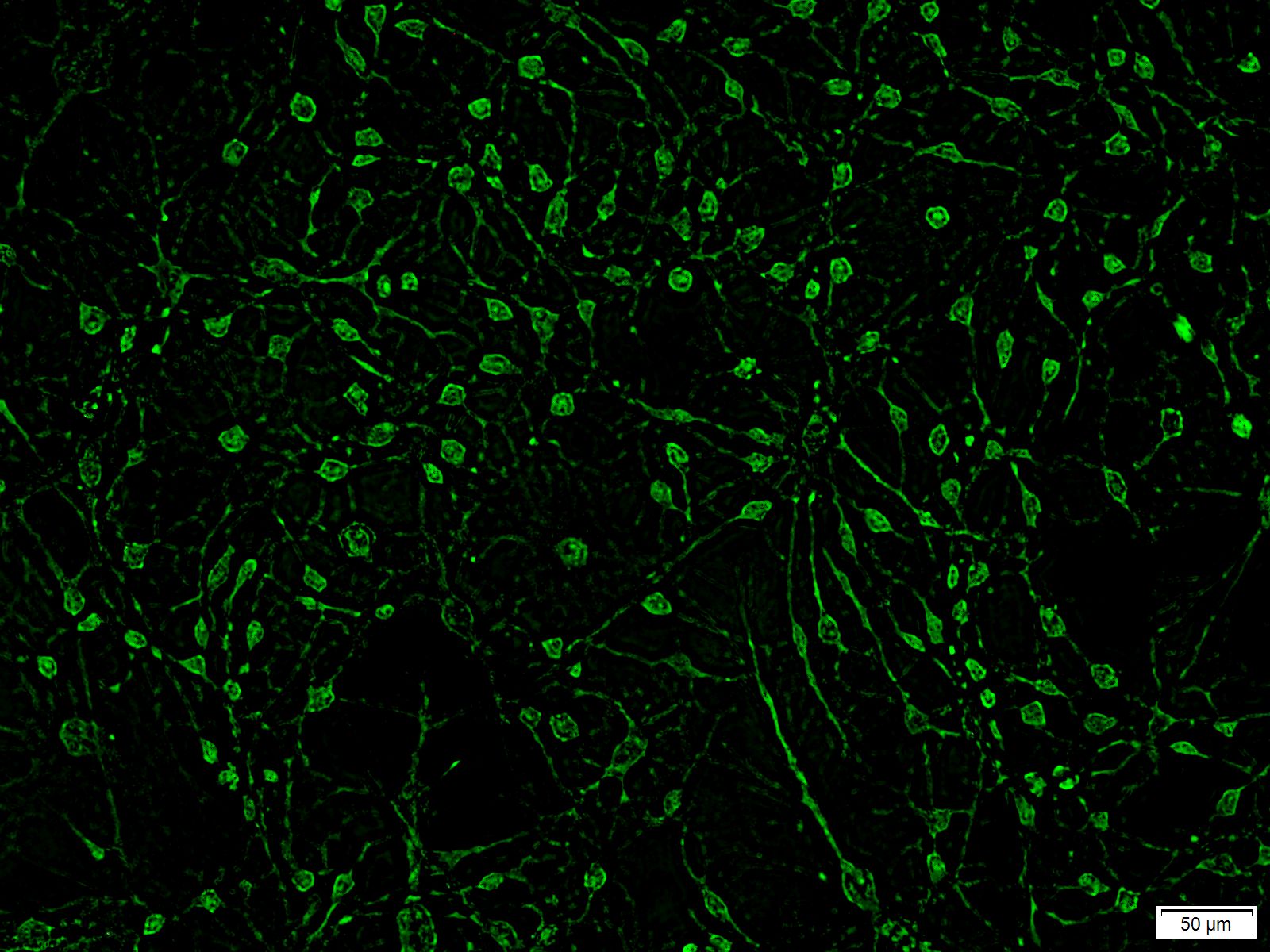

Supplement: Figure S3 — Raw data: Figure 3 A-F [file peerj-11-14838-s020.zip › Figure 4/A/control(2).jpg]

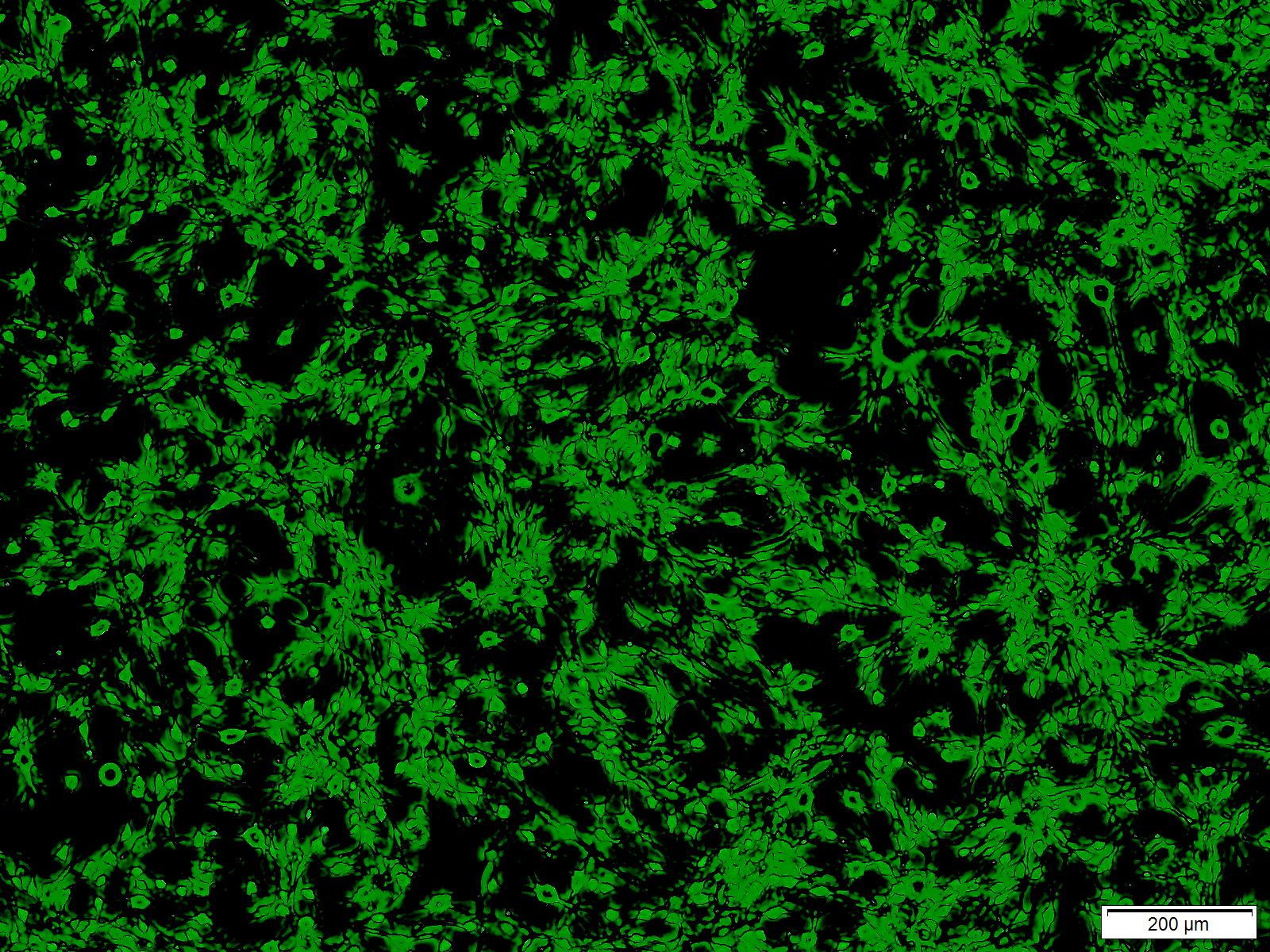

Supplement: Figure S3 — Raw data: Figure 3 A-F [file peerj-11-14838-s020.zip › Figure 4/A/inhabitor (1).jpg]

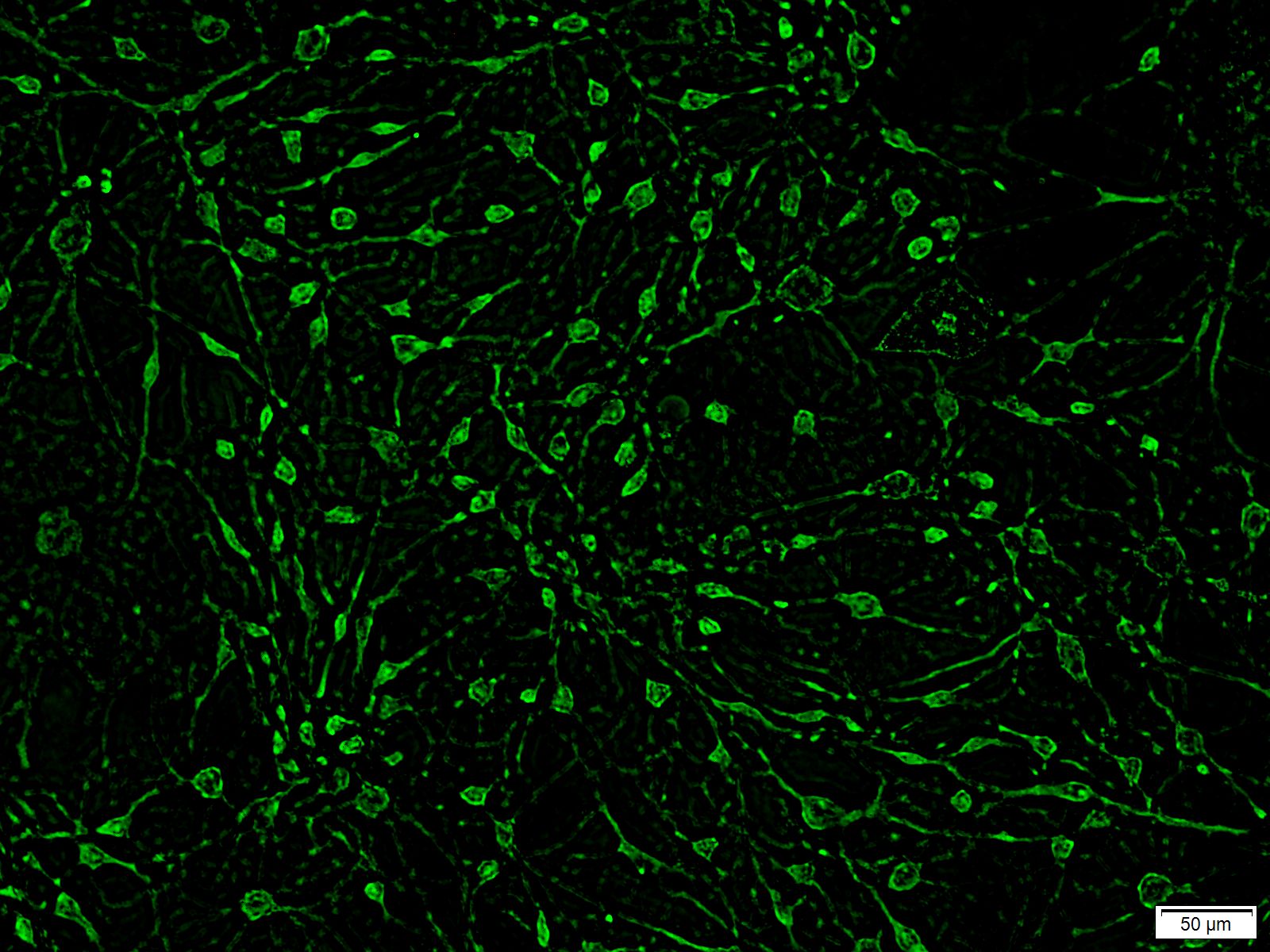

Supplement: Figure S3 — Raw data: Figure 3 A-F [file peerj-11-14838-s020.zip › Figure 4/A/inhabitor (2).jpg]

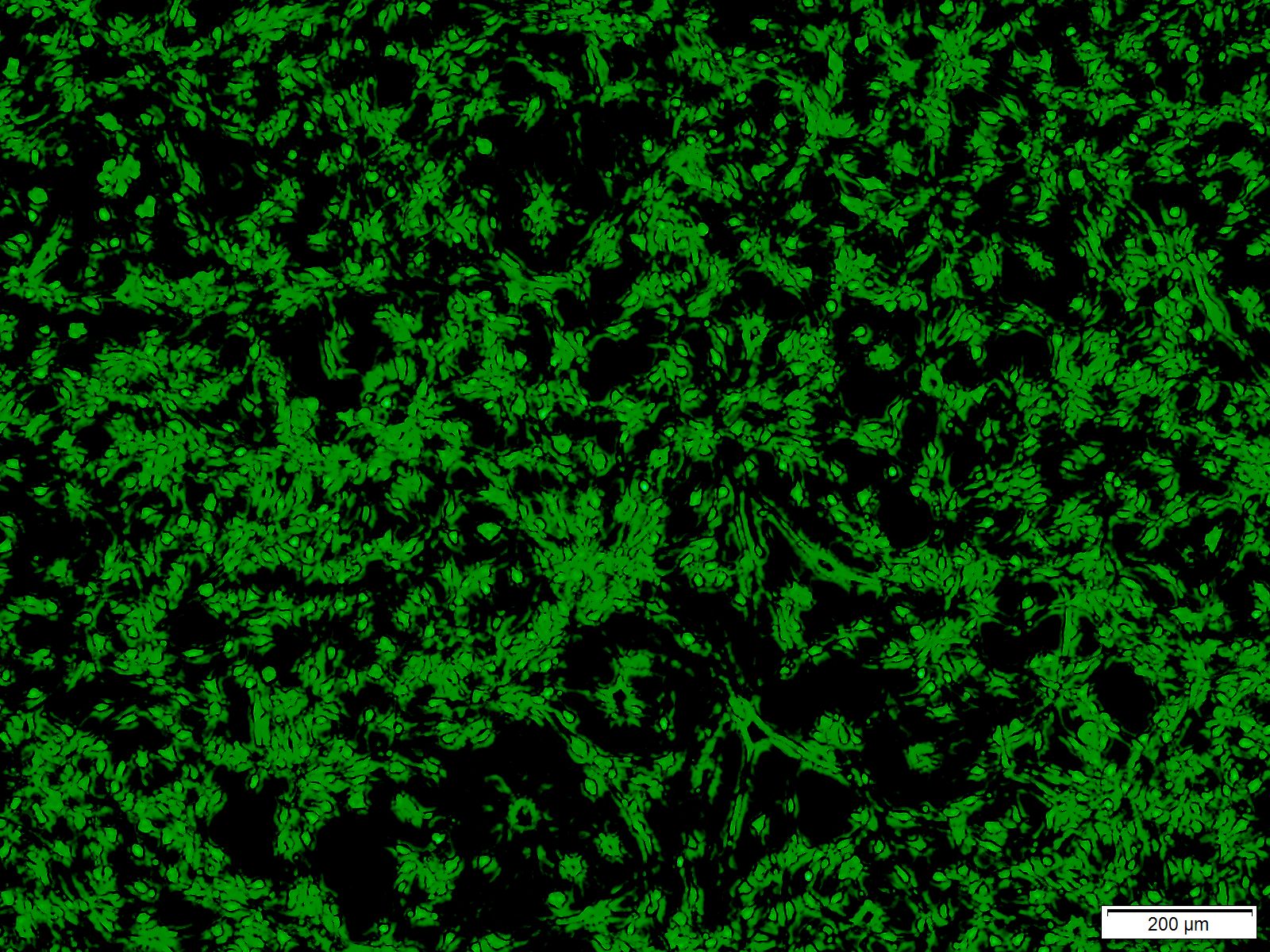

Supplement: Figure S3 — Raw data: Figure 3 A-F [file peerj-11-14838-s020.zip › Figure 4/A/mimics(1).jpg]

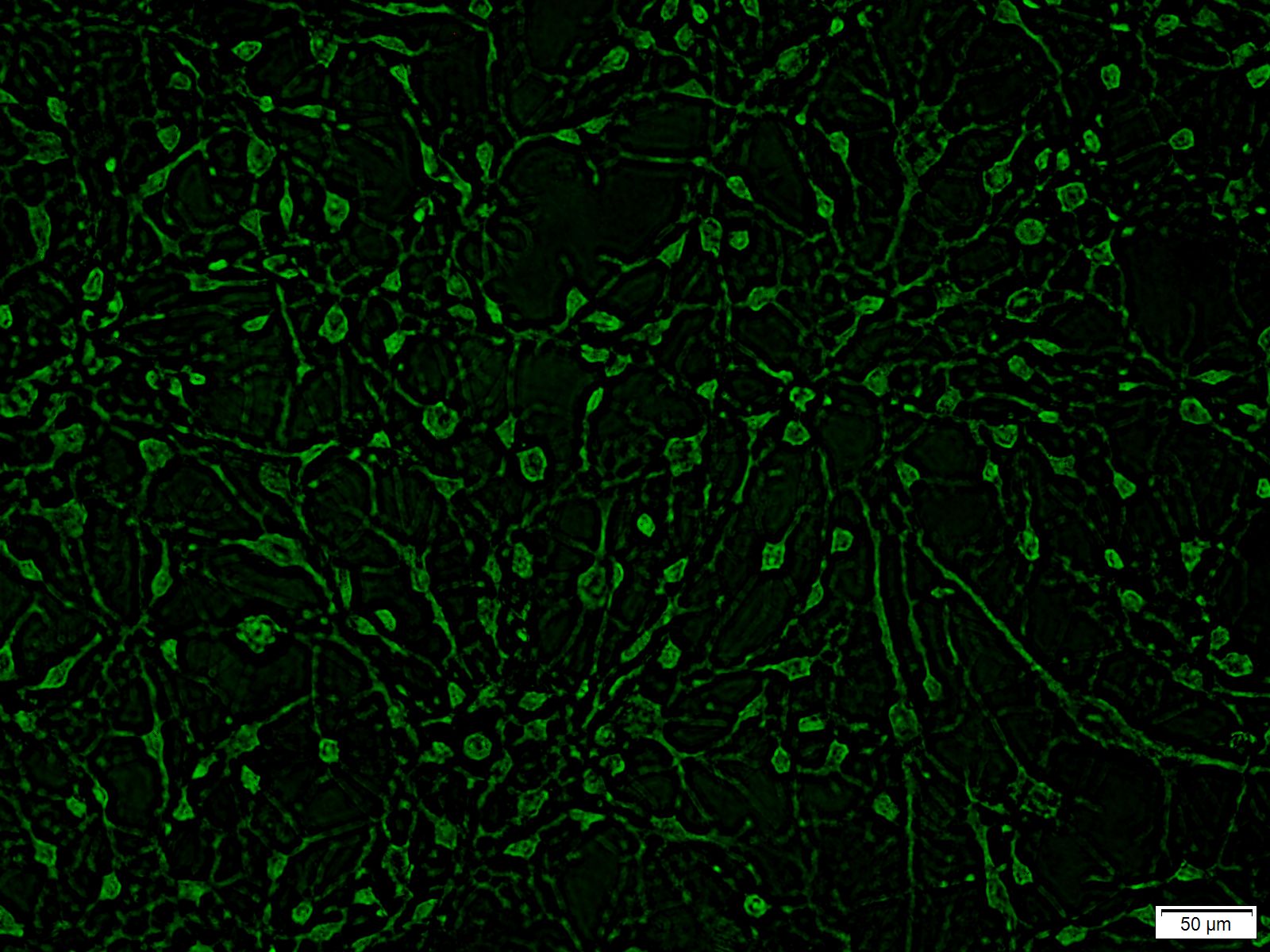

Supplement: Figure S3 — Raw data: Figure 3 A-F [file peerj-11-14838-s020.zip › Figure 4/A/mimics(2).jpg]

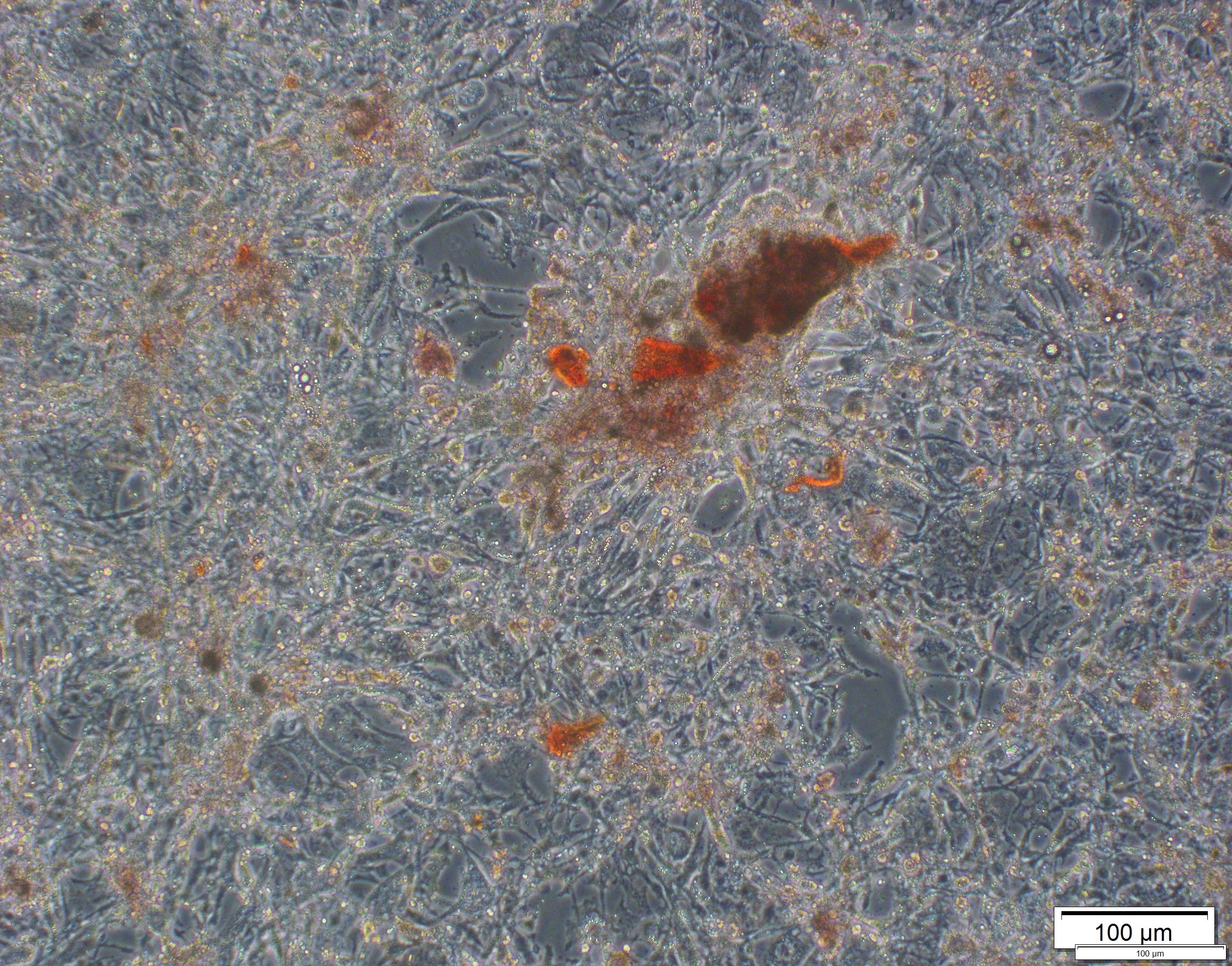

Supplement: Figure S3 — Raw data: Figure 3 A-F [file peerj-11-14838-s020.zip › Figure 4/C/control(1).jpg]

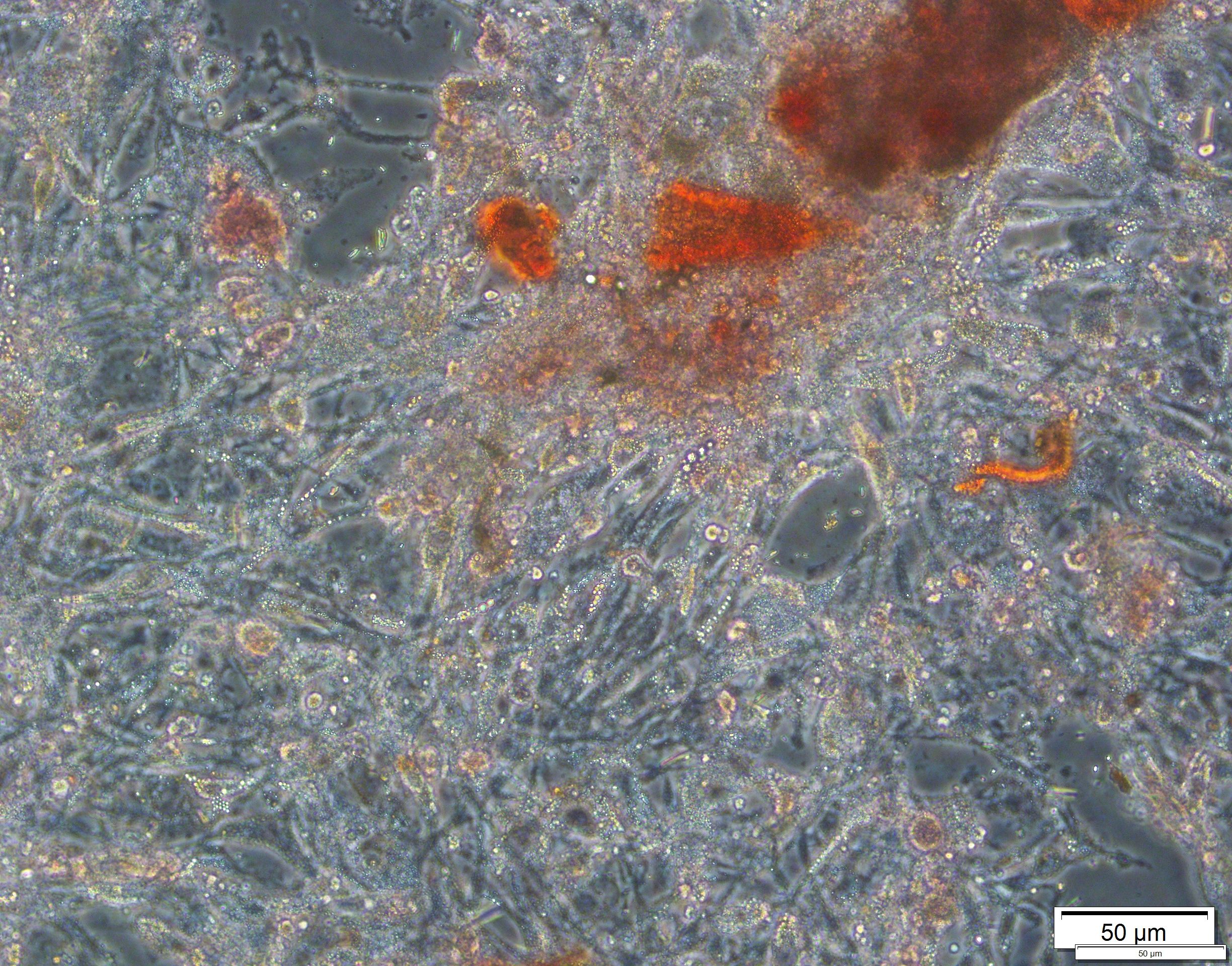

Supplement: Figure S3 — Raw data: Figure 3 A-F [file peerj-11-14838-s020.zip › Figure 4/C/control(2).jpg]

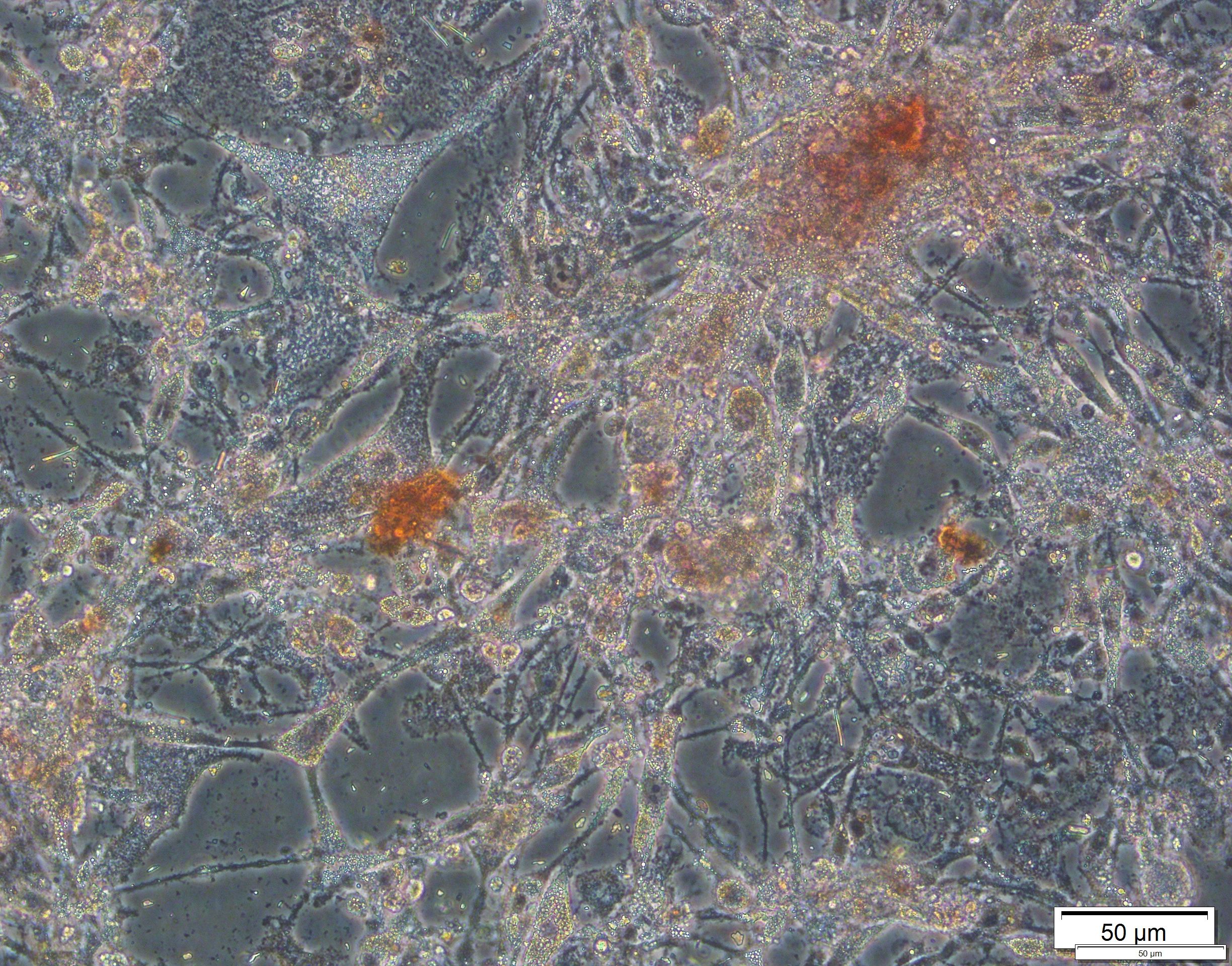

Supplement: Figure S3 — Raw data: Figure 3 A-F [file peerj-11-14838-s020.zip › Figure 4/C/inhabitor (2).jpg]

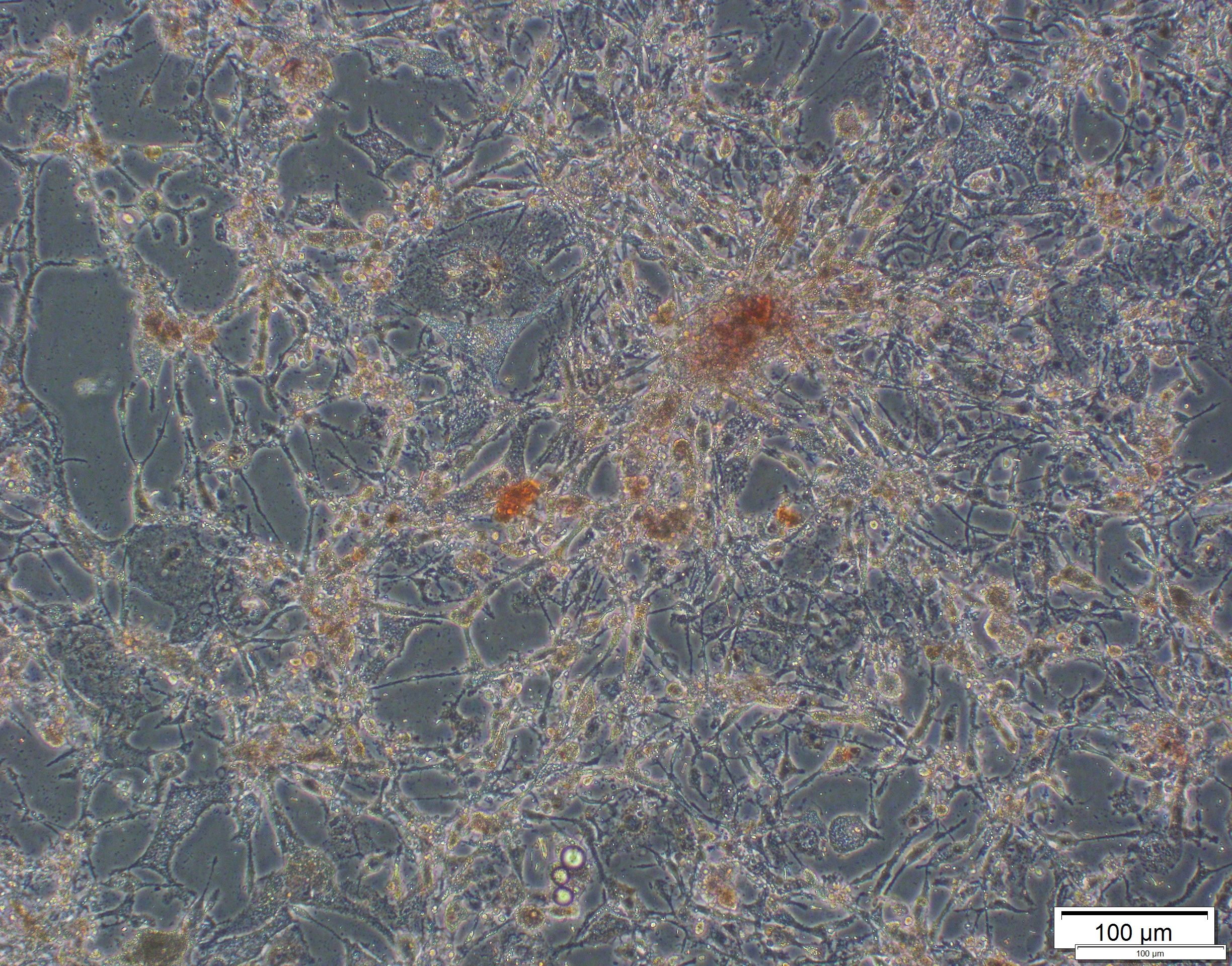

Supplement: Figure S3 — Raw data: Figure 3 A-F [file peerj-11-14838-s020.zip › Figure 4/C/inhabitor(1).jpg]

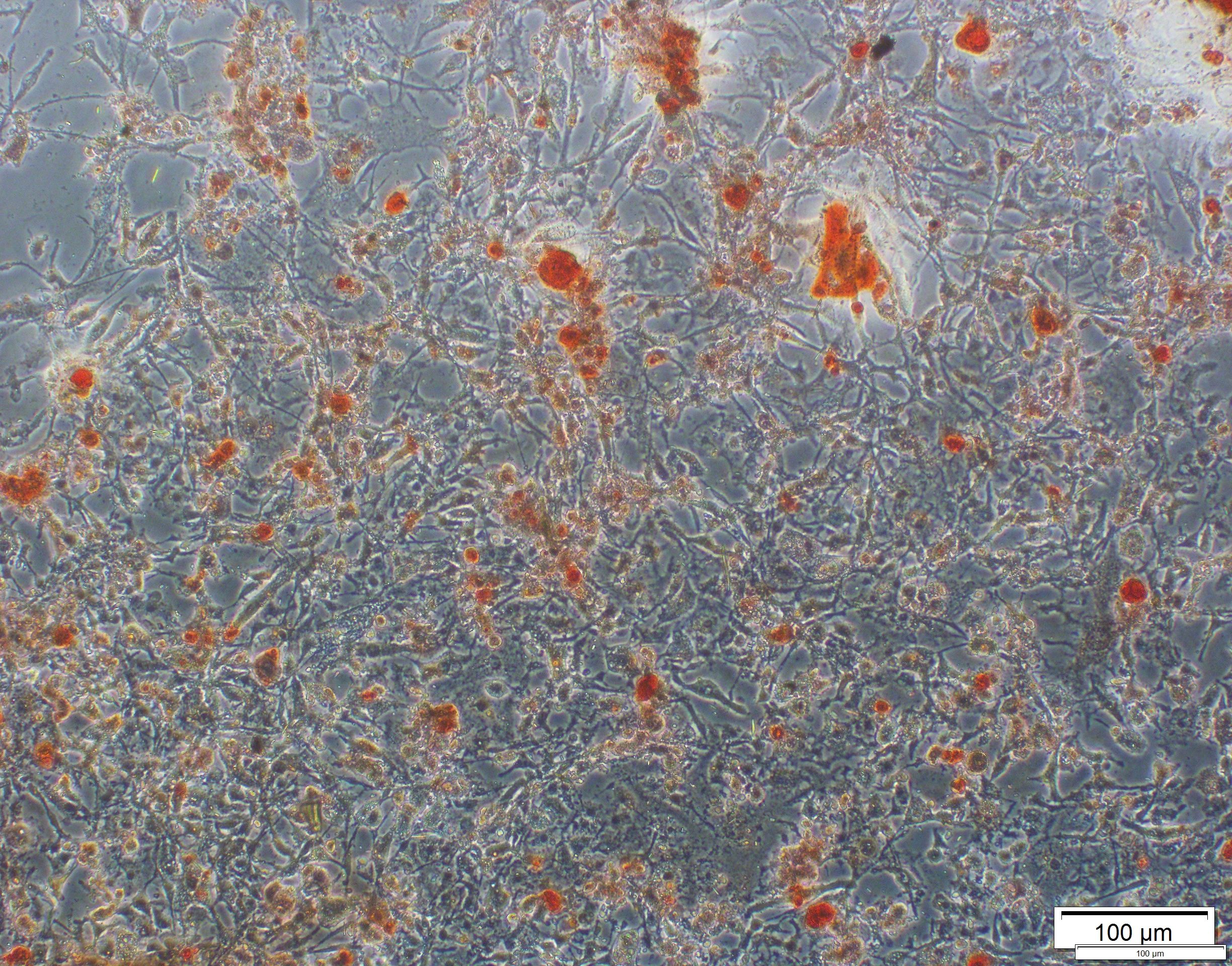

Supplement: Figure S3 — Raw data: Figure 3 A-F [file peerj-11-14838-s020.zip › Figure 4/C/mimics(1).jpg]

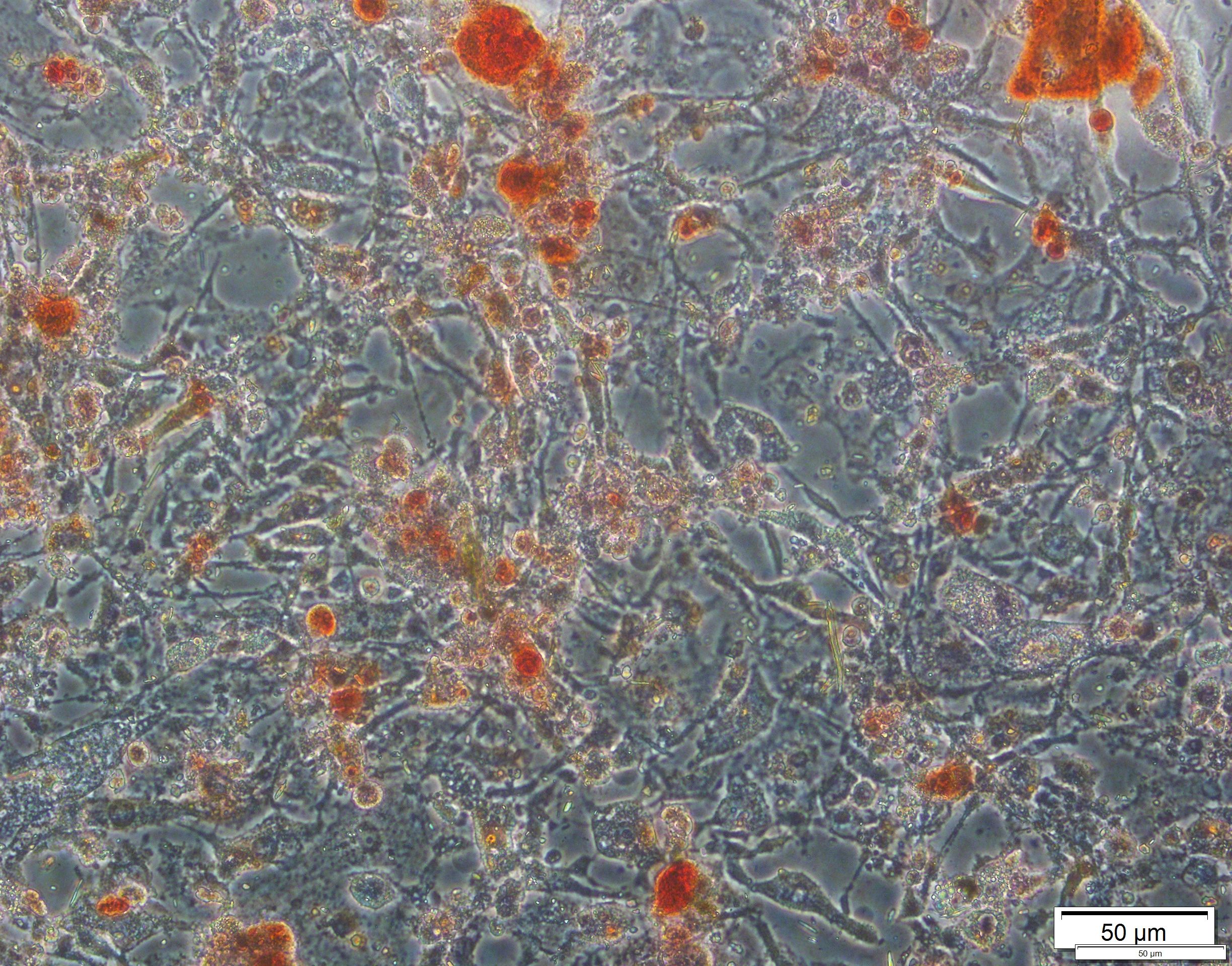

Supplement: Figure S3 — Raw data: Figure 3 A-F [file peerj-11-14838-s020.zip › Figure 4/C/mimics(2).jpg]

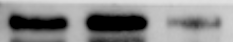

Supplement: Figure S3 — Raw data: Figure 3 A-F [file peerj-11-14838-s020.zip › Figure 4/E/screenshots/RUNX2.png]

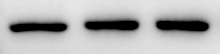

Supplement: Figure S3 — Raw data: Figure 3 A-F [file peerj-11-14838-s020.zip › Figure 4/E/screenshots/β-actin.png]

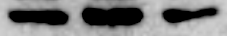

Supplement: Figure S3 — Raw data: Figure 3 A-F [file peerj-11-14838-s020.zip › Figure 4/F/screenshots/Runx2.png]

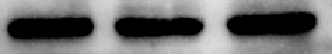

Supplement: Figure S3 — Raw data: Figure 3 A-F [file peerj-11-14838-s020.zip › Figure 4/F/screenshots/β-actin.png]

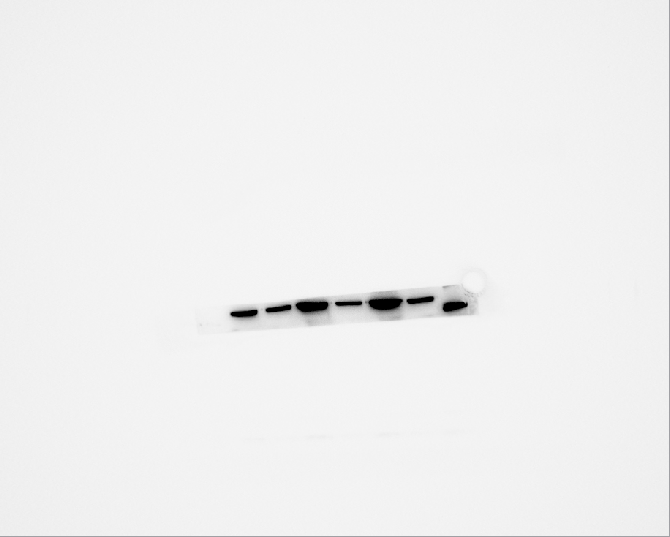

Supplement: Figure S4 — Raw data: Figure 4 A-F [file peerj-11-14838-s021.zip › Figure 5/F/PPAR-a├,line1-3]

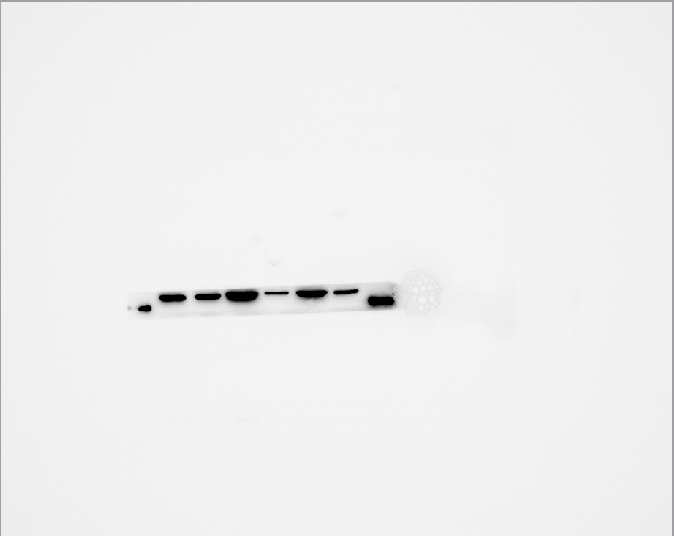

Supplement: Figure S4 — Raw data: Figure 4 A-F [file peerj-11-14838-s021.zip › Figure 5/E/1649987982(1).jpg]

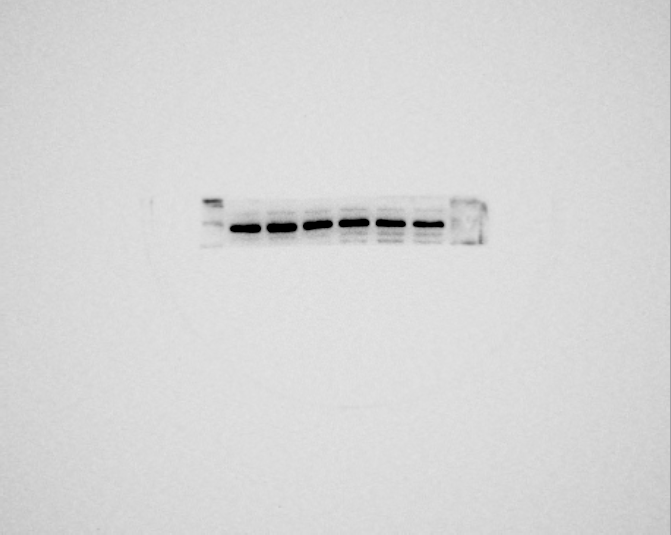

Supplement: Figure S4 — Raw data: Figure 4 A-F [file peerj-11-14838-s021.zip › Figure 5/F/a┬-actin,line1-3]

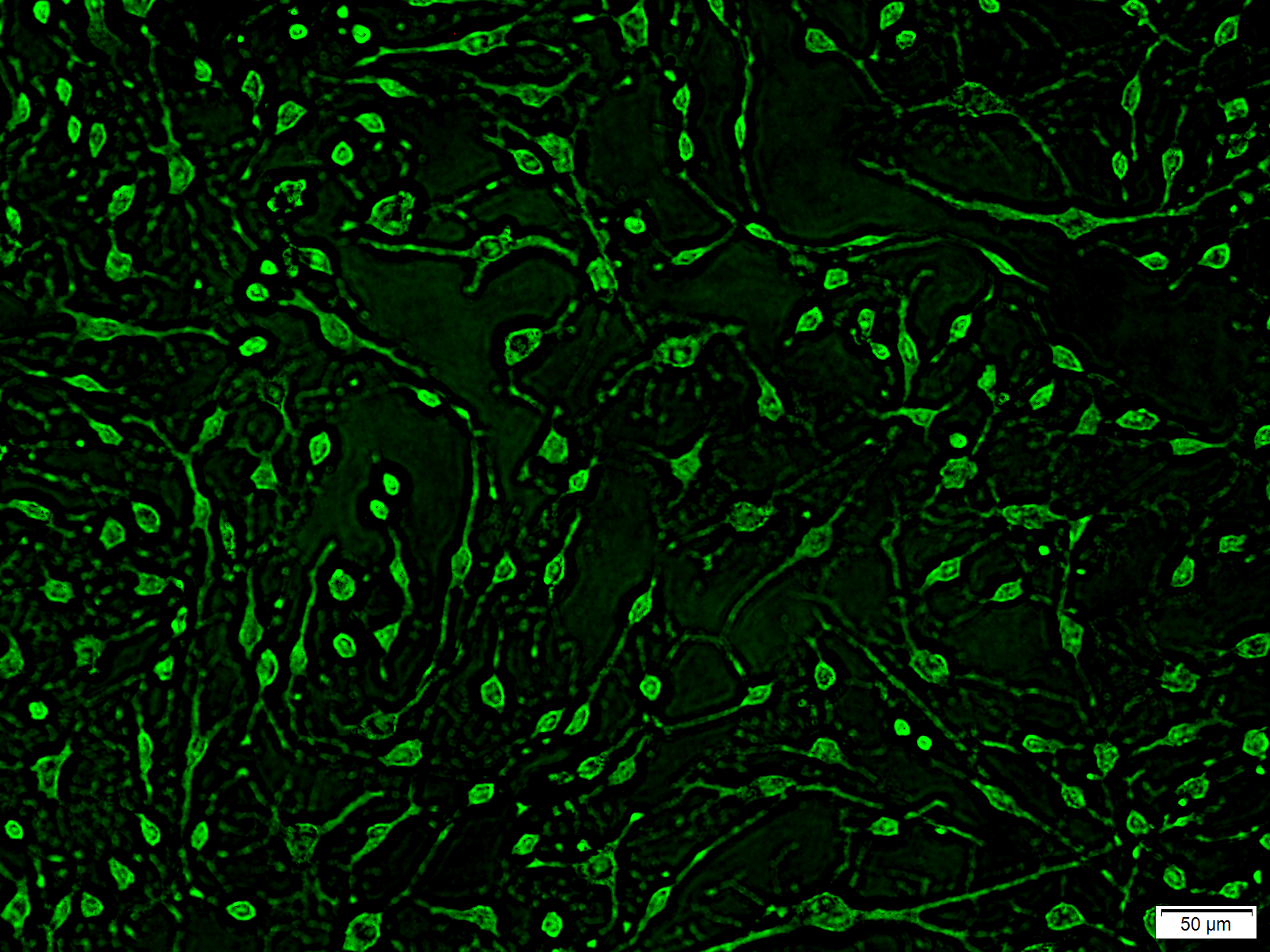

Supplement: Figure S4 — Raw data: Figure 4 A-F [file peerj-11-14838-s021.zip › Figure 5/A/contorl (1).tif]

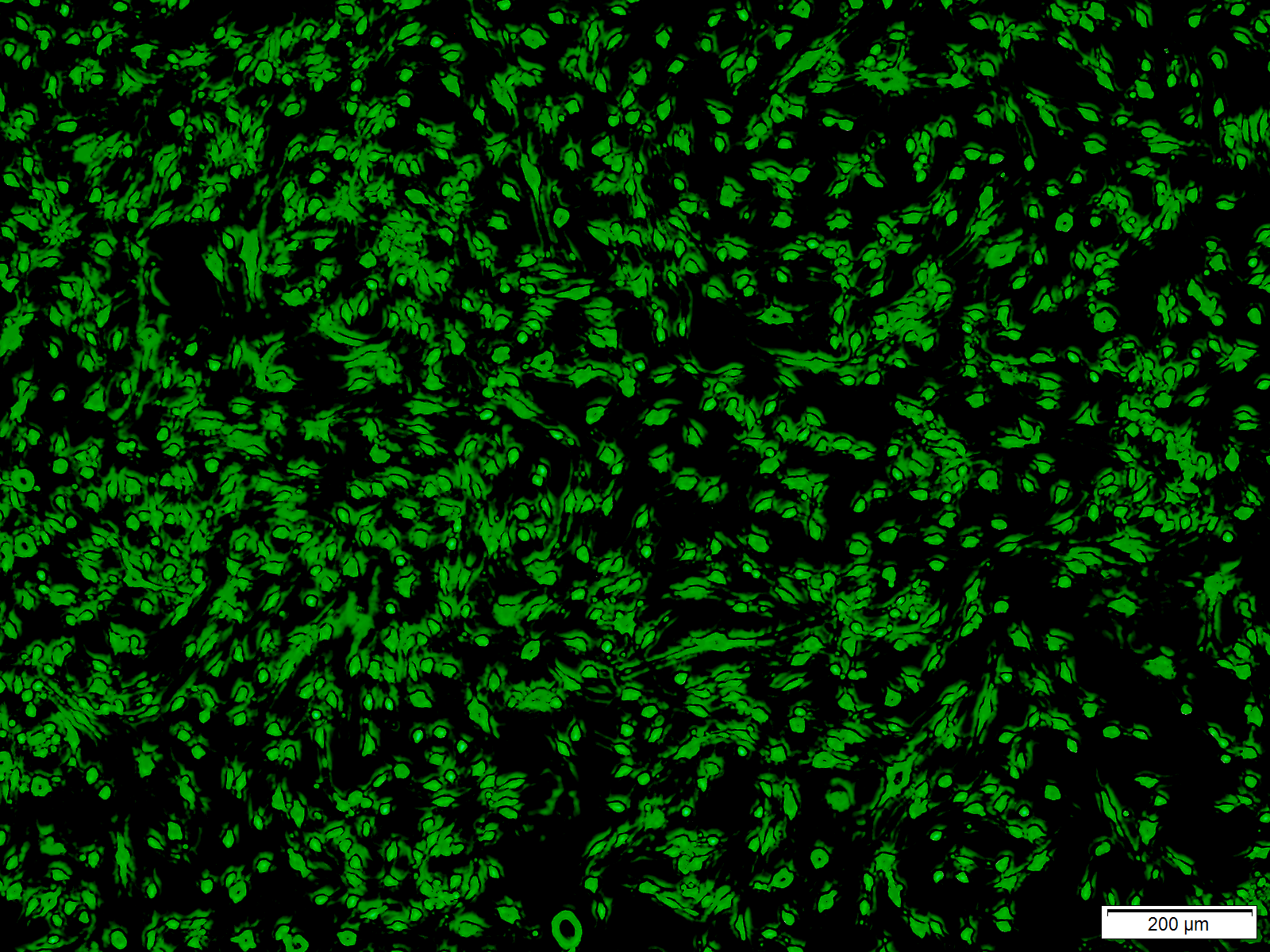

Supplement: Figure S4 — Raw data: Figure 4 A-F [file peerj-11-14838-s021.zip › Figure 5/A/contorl (2).tif]

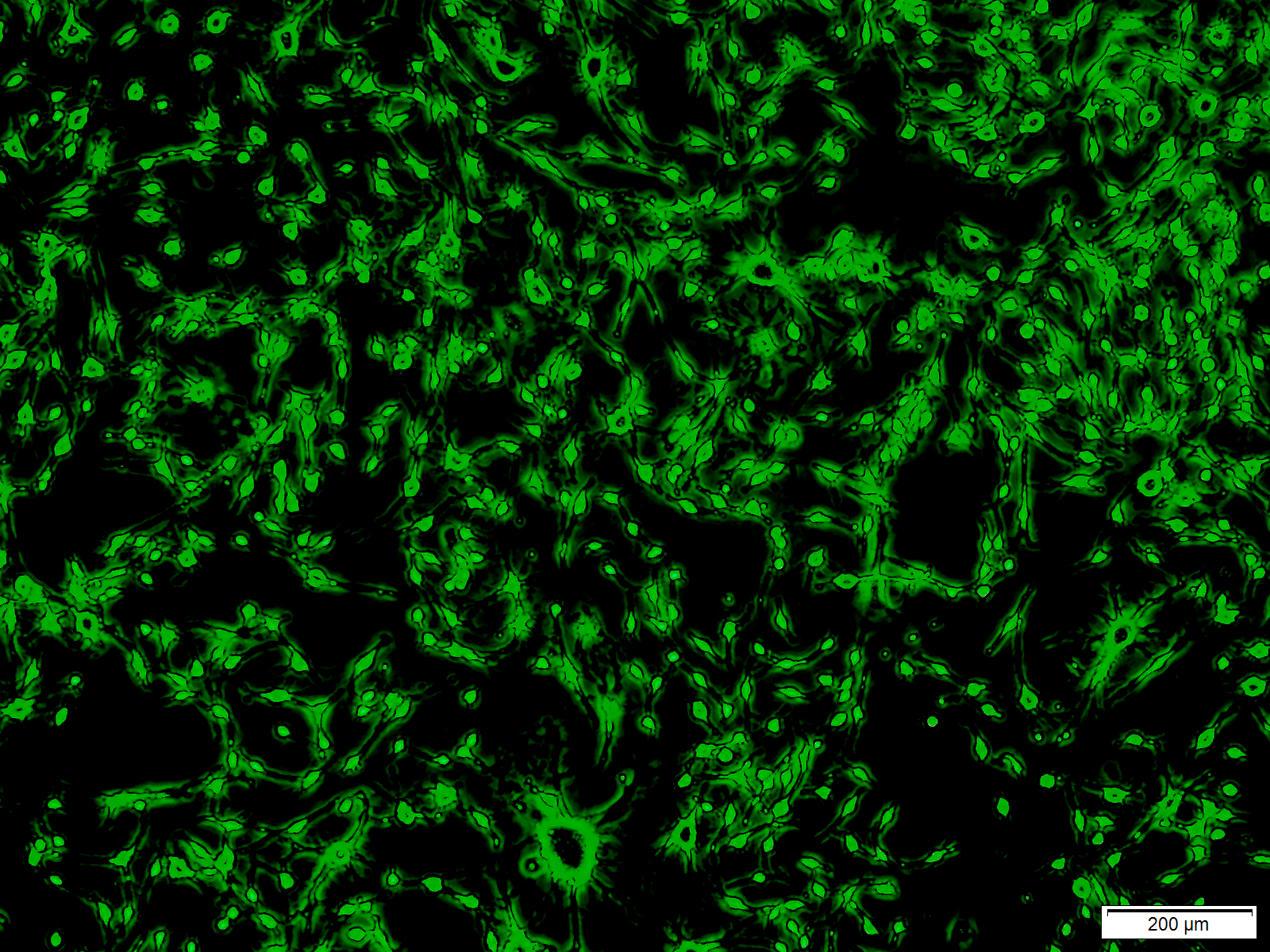

Supplement: Figure S4 — Raw data: Figure 4 A-F [file peerj-11-14838-s021.zip › Figure 5/A/inhabitor (1).tif]

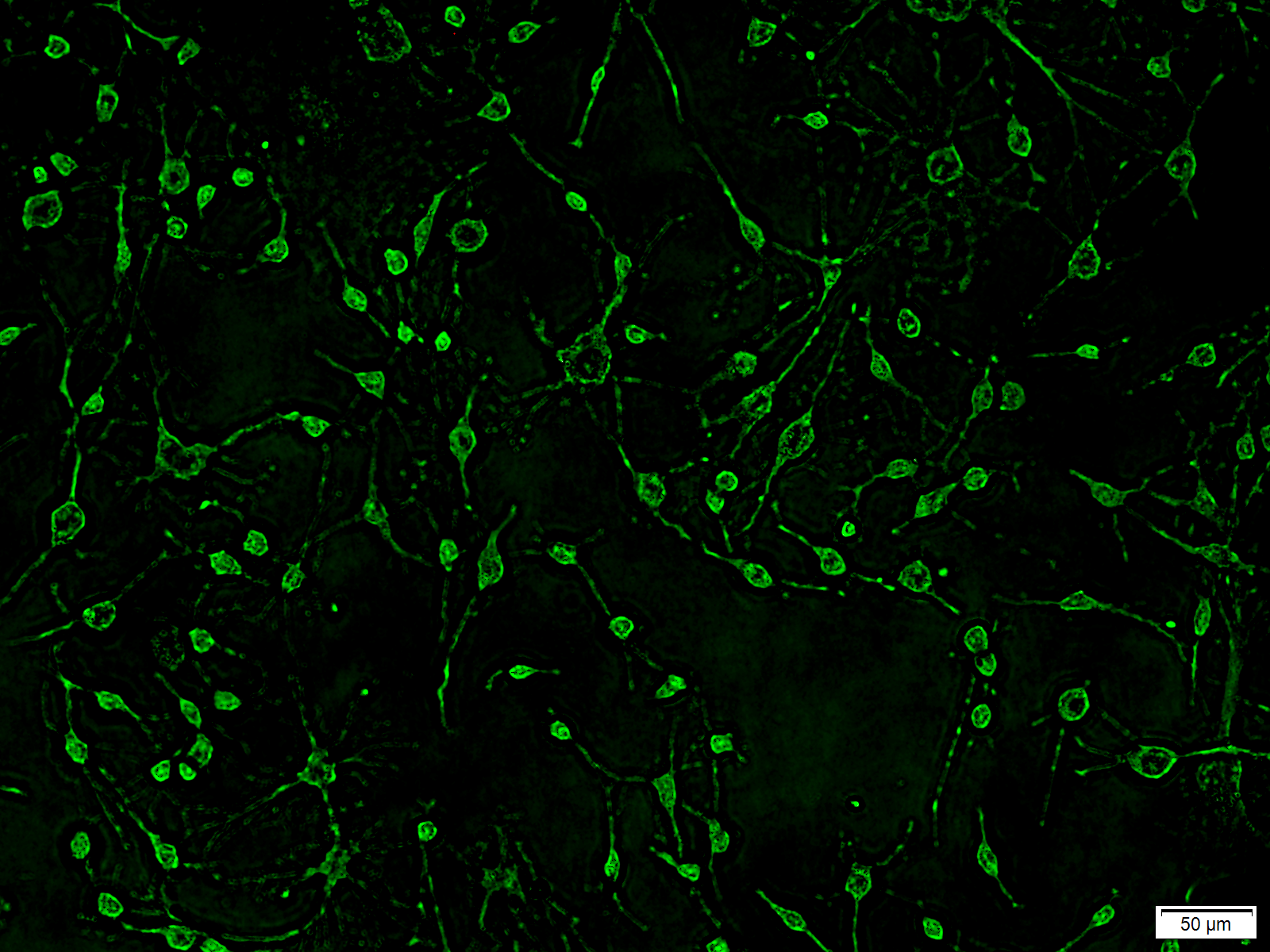

Supplement: Figure S4 — Raw data: Figure 4 A-F [file peerj-11-14838-s021.zip › Figure 5/A/inhabitor (2).tif]

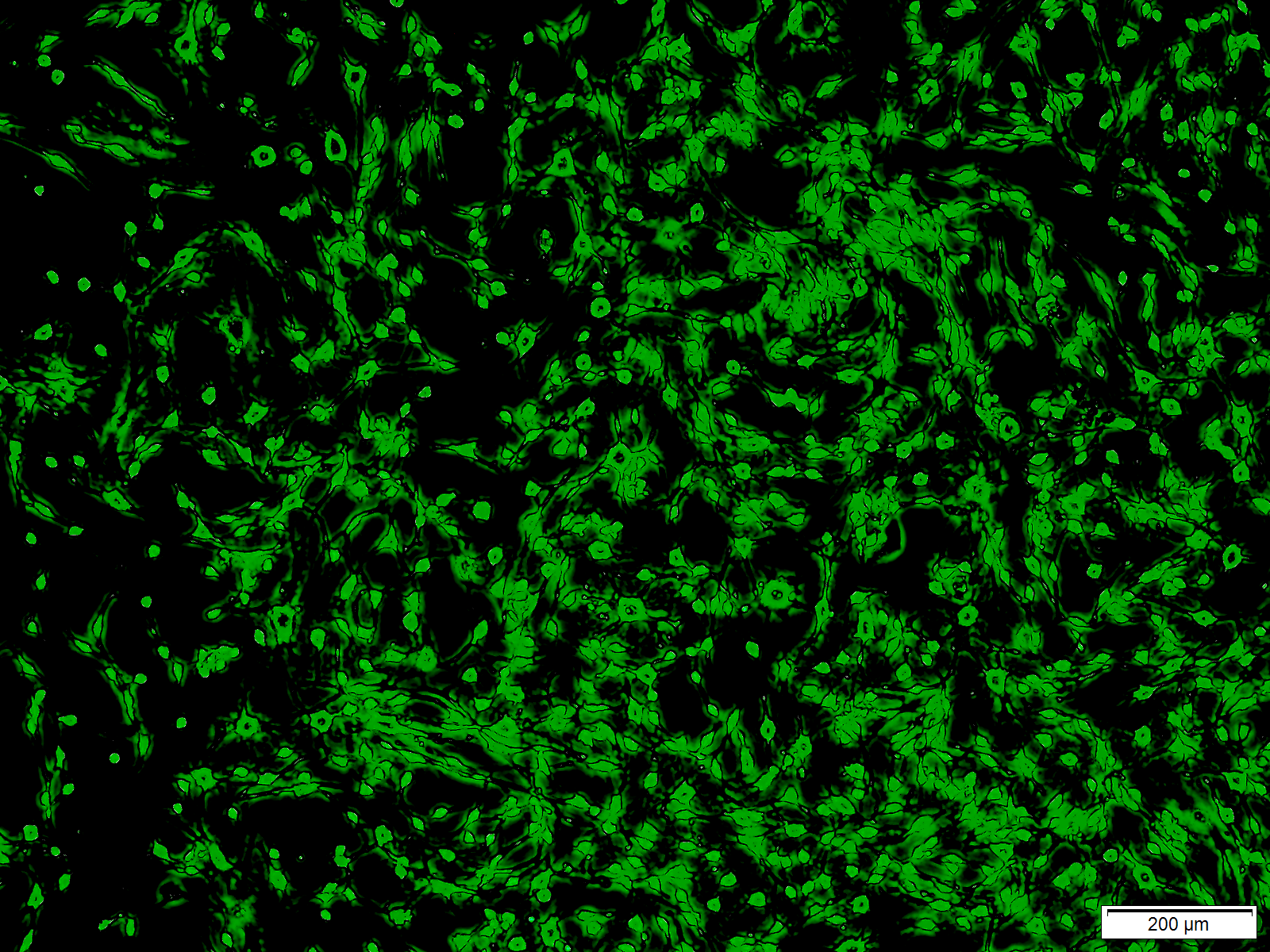

Supplement: Figure S4 — Raw data: Figure 4 A-F [file peerj-11-14838-s021.zip › Figure 5/A/mimics (1).tif]

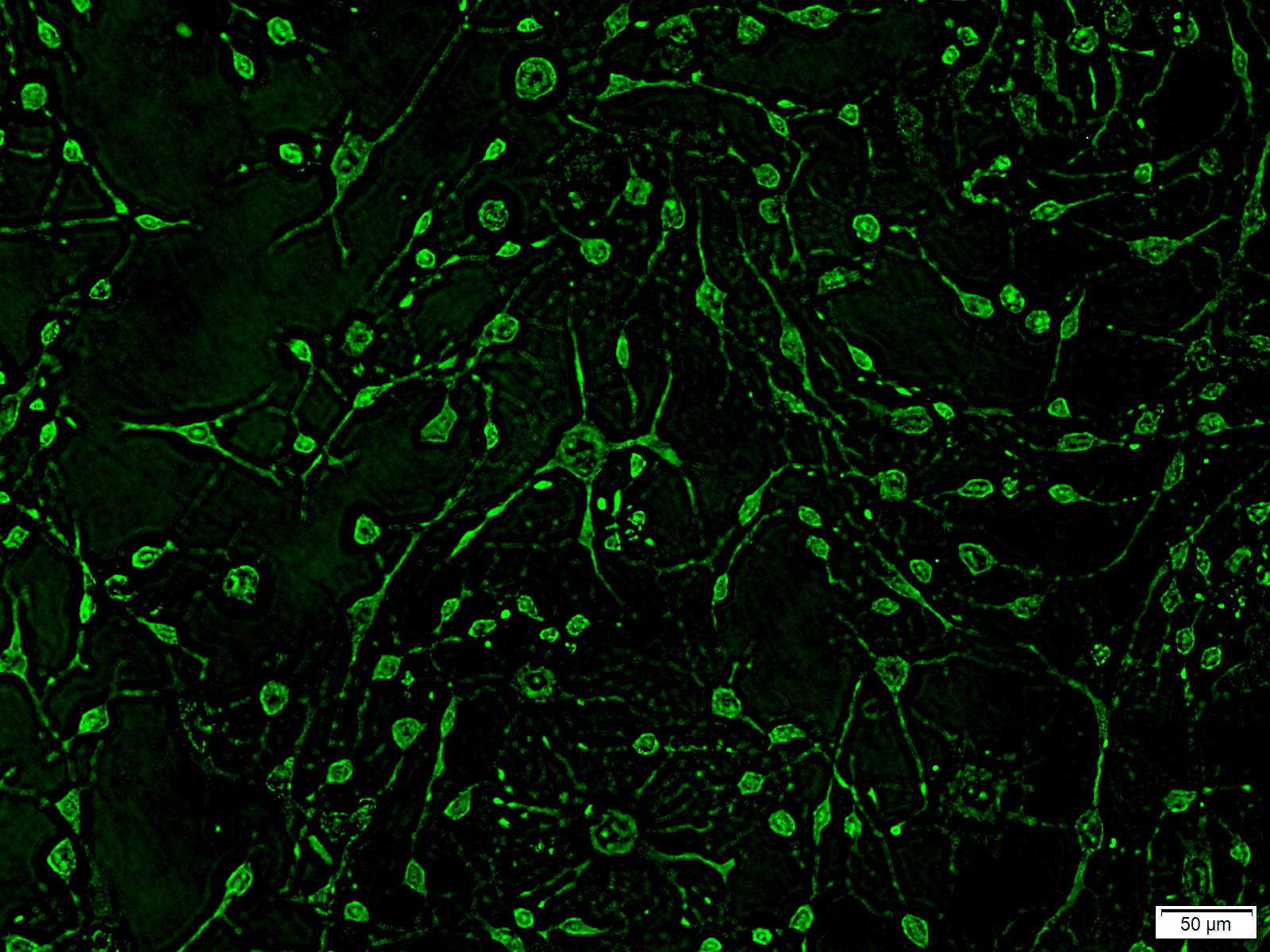

Supplement: Figure S4 — Raw data: Figure 4 A-F [file peerj-11-14838-s021.zip › Figure 5/A/mimics (2).tif]

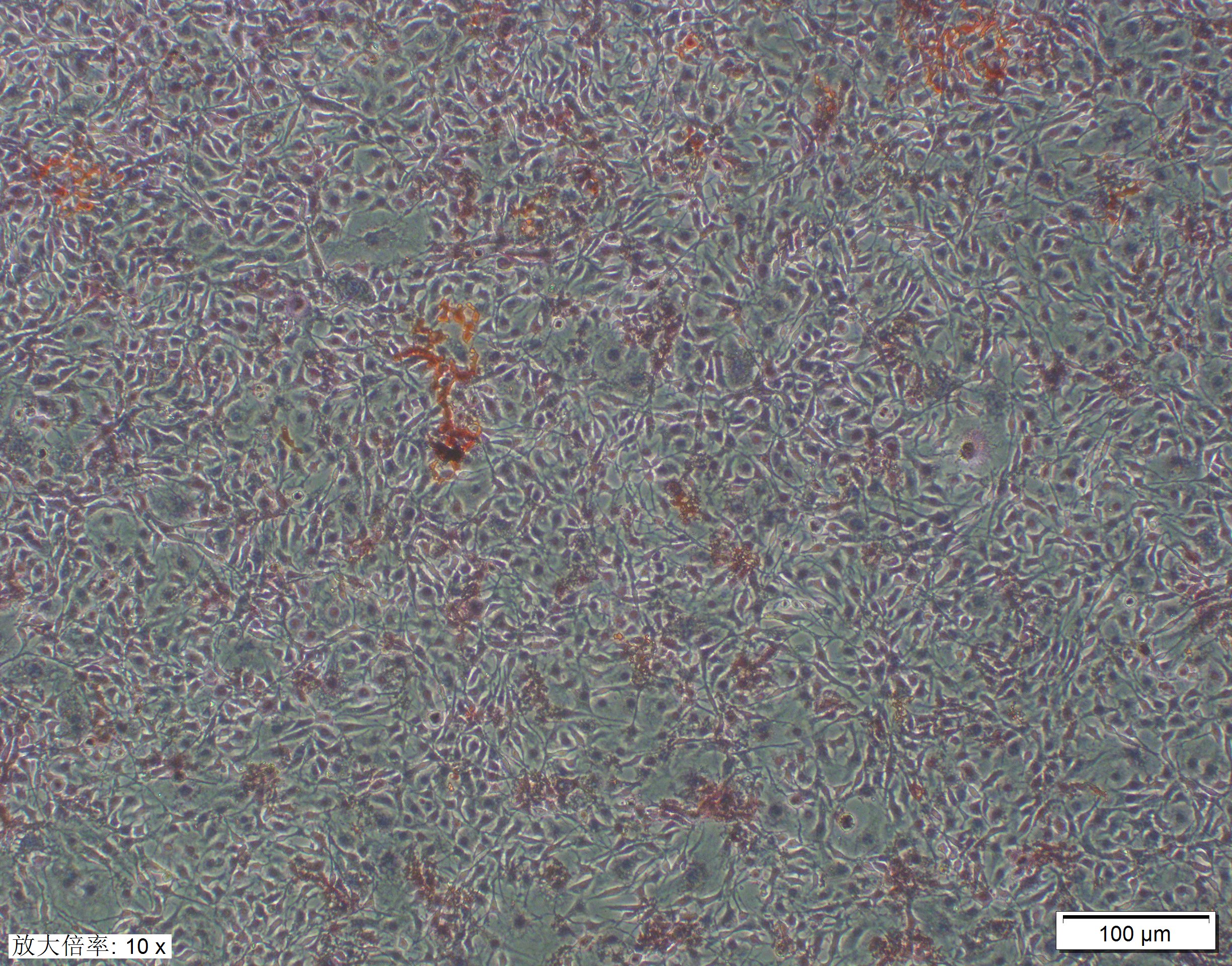

Supplement: Figure S4 — Raw data: Figure 4 A-F [file peerj-11-14838-s021.zip › Figure 5/C/control(1).jpg]

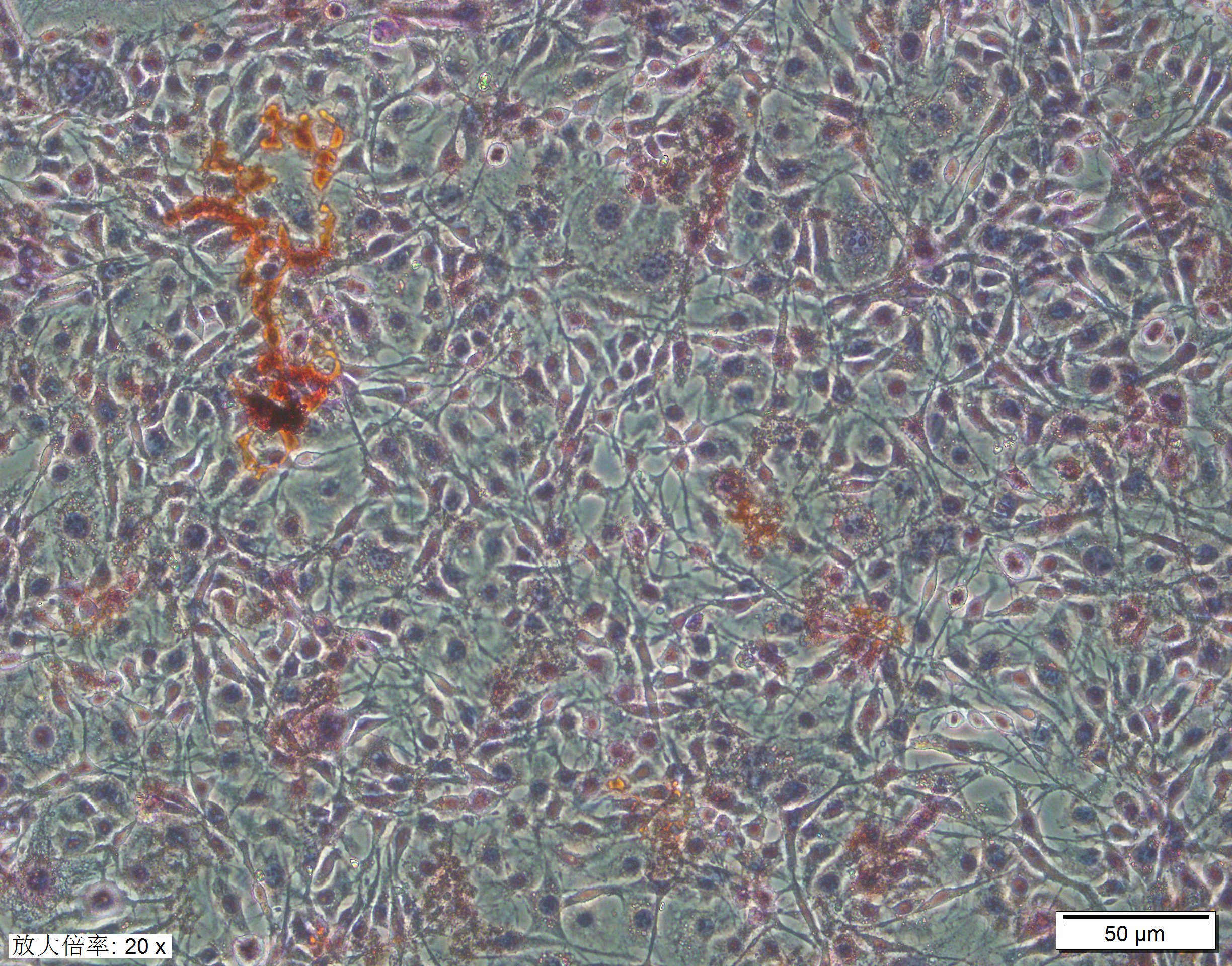

Supplement: Figure S4 — Raw data: Figure 4 A-F [file peerj-11-14838-s021.zip › Figure 5/C/control(2).jpg]

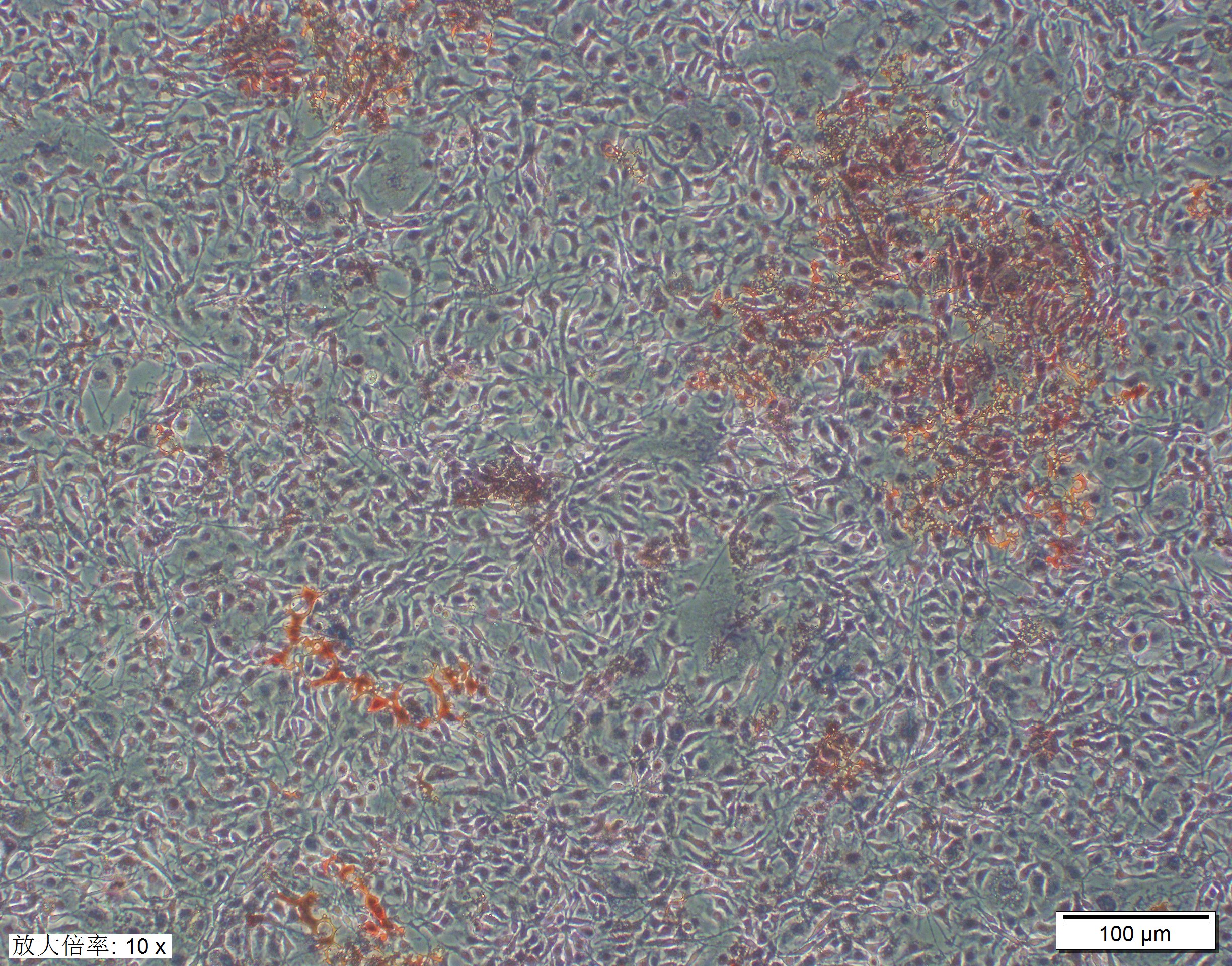

Supplement: Figure S4 — Raw data: Figure 4 A-F [file peerj-11-14838-s021.zip › Figure 5/C/inhabitor(1).jpg]

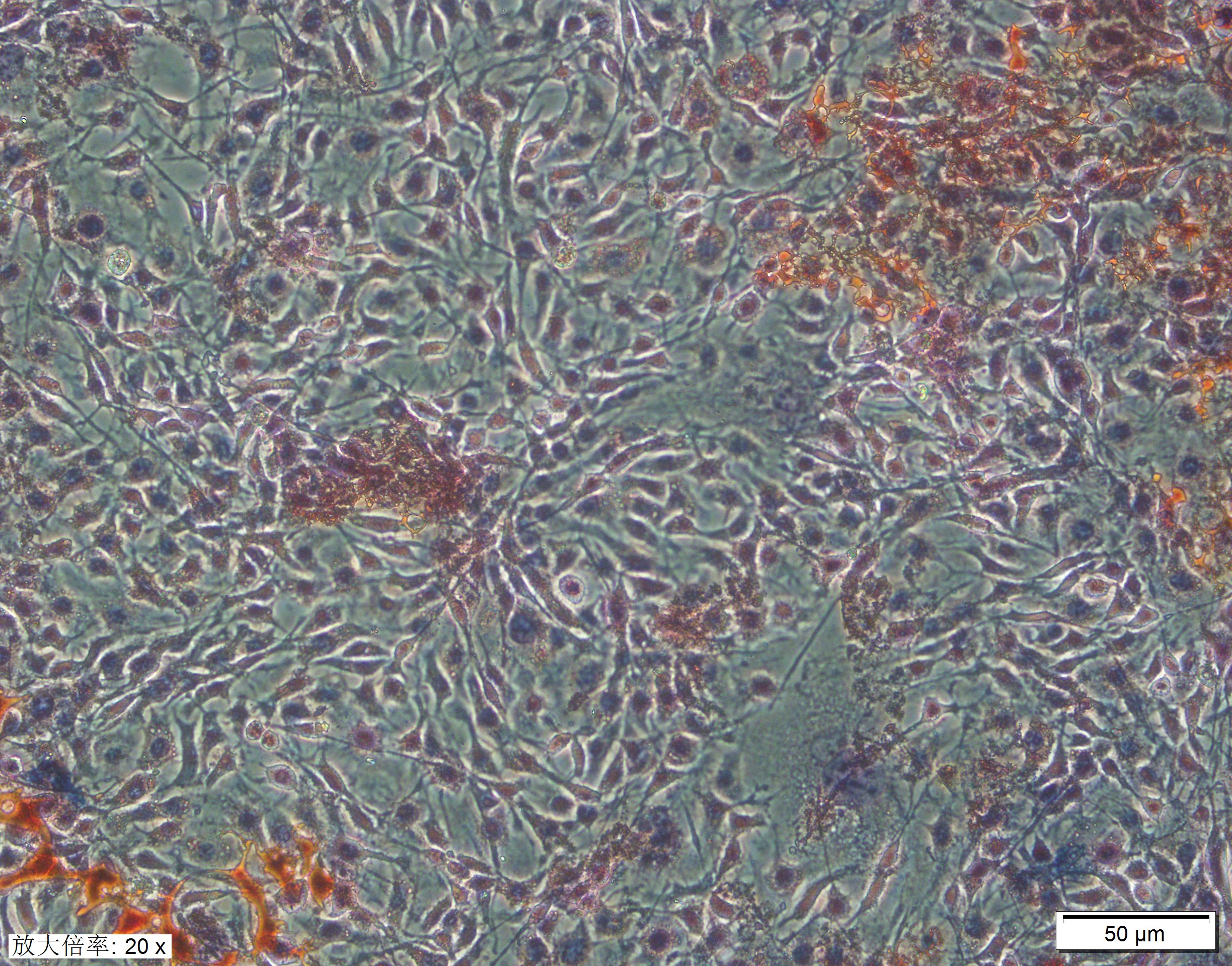

Supplement: Figure S4 — Raw data: Figure 4 A-F [file peerj-11-14838-s021.zip › Figure 5/C/inhabitor(2).jpg]

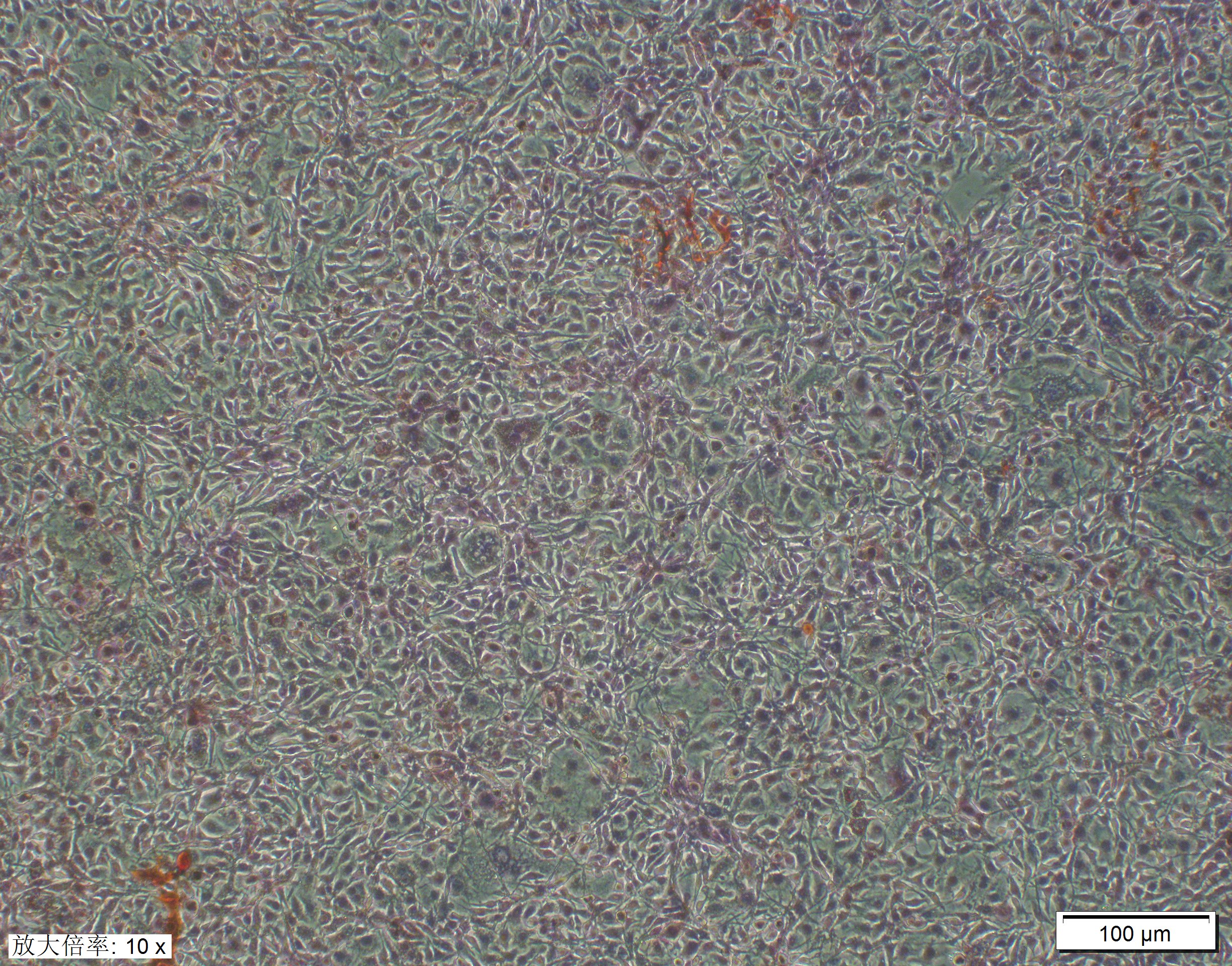

Supplement: Figure S4 — Raw data: Figure 4 A-F [file peerj-11-14838-s021.zip › Figure 5/C/mimics(1).jpg]

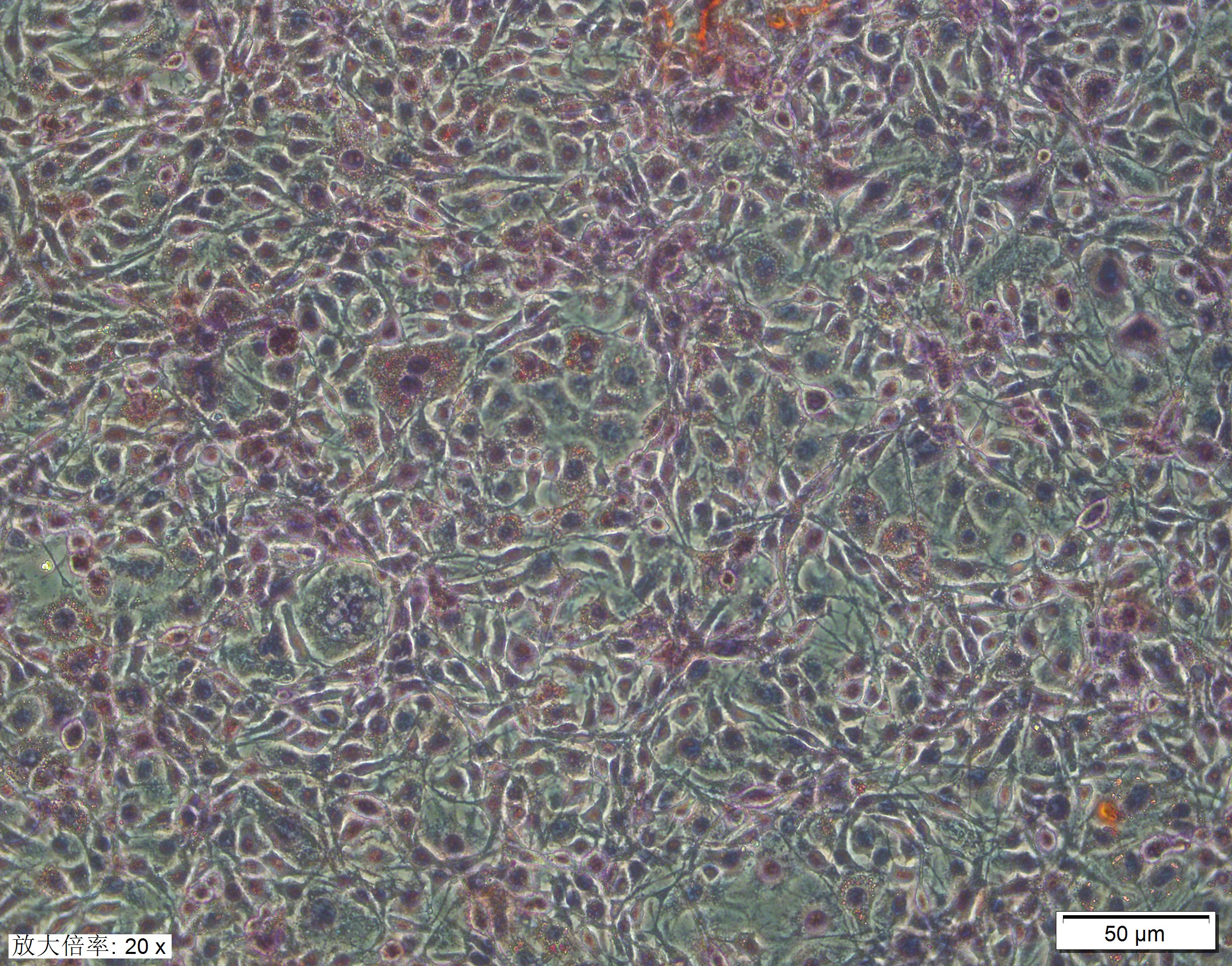

Supplement: Figure S4 — Raw data: Figure 4 A-F [file peerj-11-14838-s021.zip › Figure 5/C/mimics(2).jpg]

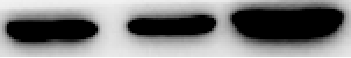

Supplement: Figure S4 — Raw data: Figure 4 A-F [file peerj-11-14838-s021.zip › Figure 5/E/screenshots/PPAR-a├.png]

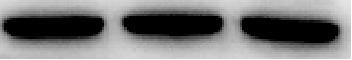

Supplement: Figure S4 — Raw data: Figure 4 A-F [file peerj-11-14838-s021.zip › Figure 5/E/screenshots/a┬-actin.png]

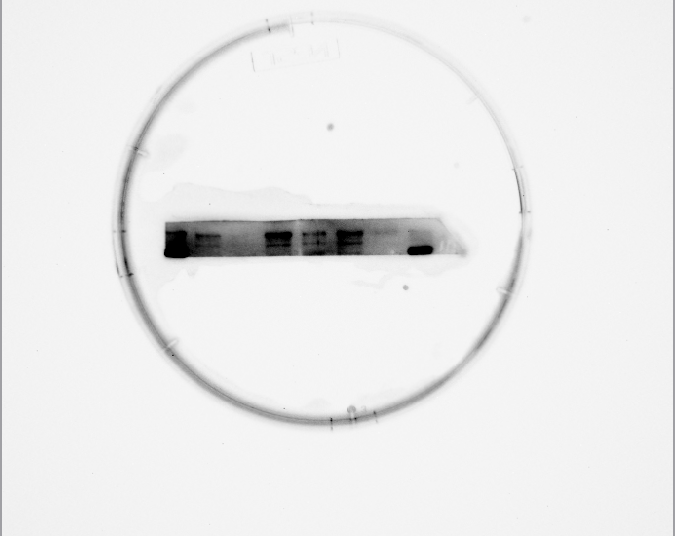

Supplement: Figure S5 — Raw data: Figure 5 A-B [file peerj-11-14838-s022.zip › Figure 6/B/Dnmt3a,line1-3]

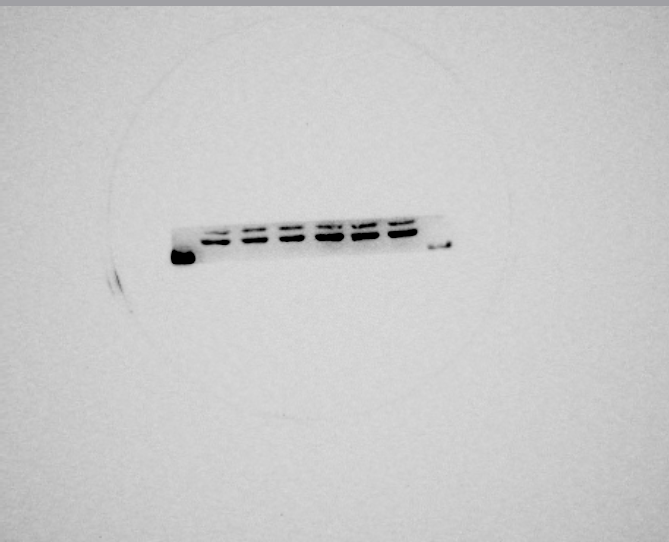

Supplement: Figure S5 — Raw data: Figure 5 A-B [file peerj-11-14838-s022.zip › Figure 6/B/a┬-actin,line1-3]

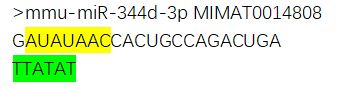

Supplement: Figure S5 — Raw data: Figure 5 A-B [file peerj-11-14838-s022.zip › Figure 6/A/screenshots.png]

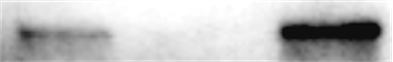

Supplement: Figure S5 — Raw data: Figure 5 A-B [file peerj-11-14838-s022.zip › Figure 6/B/screenshots/dnmt3a.png]

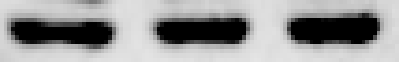

Supplement: Figure S5 — Raw data: Figure 5 A-B [file peerj-11-14838-s022.zip › Figure 6/B/screenshots/a┬-actin.png]
