# Supplementary material for: miR-344d-3p regulates osteogenic and adipogenic differentiation of mouse mandibular bone marrow mesenchymal stem cells
Source: PeerJ. 2023 Feb 14;11:e14838. doi: 10.7717/peerj.14838 (PMC9936866; doi:10.7717/peerj.14838)

>mmu-miR-344d-3p MIMAT0014808

GAUAUAACCACUGCCAGACUGA

GTTATAT

GTTATA

TTATAT

Two 6-binding sites were manually predicted (green)

3‘ UTR of Dnmt3a

Mus musculus DNA methyltransferase 3A (Dnmt3a), transcript variant 1, mRNA

NCBI Reference Sequence: NM_007872.4

gggacatgggggcaaactgaagtagtgatgataaaaaagttaaacaaacaaacaaacaaaaaacaaaacaaaacaataaaacaccaagaacgagaggacggagaaaagttcagcacccagaagagaaaaaggaatttaaagcaaaccacagaggaggaaaacgccggagggcttggccttgcaaaagggttggacatcatctcctgagttttcaatgttaaccttcagtcctatctaaaaagcaaaataggcccctccccttcttcccctccggtcctaggaggcgaactttttgttttctactctttttcagaggggttttctgtttgtttgggtttttgtttcttgctgtgactgaaacaagagagttattgcagcaaaatcagtaacaacaaaaagtagaaatgccttggagaggaaagggagagagggaaaattctataaaaacttaaaatattggtttttttttttttccttttctatatatctctttggttgtctctagcctgatcagataggagcacaaacaggaagagaatagagaccctcggaggcagagtctcctctcccaccccccgagcagtctcaacagcaccattcctggtcatgcaaaacagaacccaactagcagcagggcgctgagagaacaccacaccagacacttttctacagtatttcaggtgcctaccacacaggaaaccttgaagaaaaccagtttctagaagccgctgttacctcttgtttacagtttatatatatatgatagatatgagatatatatatataaaaggtactgttaactactgtacatcccgacttcataatggtgctttcaaaacagcgagatgagcaaagacatcagcttccgcctggccctctgtgcaaagggtttcagcccaggatggggagaggggagcagctggagggggttttaacaaactgaaggatgacccatatcaccccccacccctgccccatgcctagcttcacctgccaaaaaggggctcagctgaggtggtcggaccctggggaagctgagtgtggaatttatccagactcgcgtgcaataaccttagaatatgaatctaaaatgactgcctcagaaaaatggcttgagaaaacattgtccctgattttgaattcgtcagccacgttgaaggccccttgtgggatcagaaatattccagagtgagggaaagtgacccgccattaaccccacctggagcaaataaaaaaacatacaaaatgtactggtgctttctgtctaagttgccttttgtgtgttcttttataaggccccaccatcccctctgcacatggcagctccggtcctggaatgtgatgtttttggtcatctctaaagactgcagtttcatacttgggaggctgatgacacctttattataattattcttatggttctggctataattgttttaagattttctttcagaaaacaaaaacccaacacccttccctttaggtttcaaaccaaggtgcgggggggtggcaggtgcttttttaaggaccagtggctctggtgccctggctcccacccctcaggccaggtgagccactgggcaacaagctaggcagccagggagtttgaggcccaccctccgggccagtcactcttctcttcttcttcccttcctcgtgagtccggtgtgtcagggctggagggaggccggggcagcctccctccttgtgtgtgtggttggagtggcgtgtgtttcttttctagtgtttgctctgatggctgtgctctcacttgagtcagcttcacctgggccatgtgctcgcactttgttccgctccccagccagggcaggcagcctggcattggcagcgggaagggaaggtgtgcttggccccagggtgagggcttgcctggatggatggagtgtagatatgtggcatatagagatatatattttataatgggaggggggacggcacctcctgggaccagcagagactgggaggtgtgctgtcaagcacatggtcccaagaccgcatccctagaaagatggggcctatgataggccctatataaatacatagatagggtcatgtataagaaatatacacagagggggttggagggagtgggctctgtgttgagcctgggtccttcccgcagacagccccacaaacagtatatcagtctaacacatgcacaggaatctatttctgtctggatgcacaccagggagcccccagcacacttctctgcaggatcaggacgatggagtcggctacacagctctctctcgtacatctgttgccctgatgacaggtagactcgatcccagaagttgctaggtgctgaagtcaggaaaaggaggaggtttgatccaagcaggtgcttcctctagcaggtctgtgttgtattccctttgcctctggtgatgtcagcttctgttagcagcctgtggttactgacctgcaactctcggcaccagtcaagtcagccctcagcccctacctattctccccaaccaaagtcgaactgaaatctctcttggccatggggcttccattgggaaaataaaatgttaaaaaaaaaattaaaatgggacagtgaacctcacttttggagtaaagcaaatcagtaattagtgctaaaactagtcagaggctttggccccttgctcactgctcgggactaagcttggctggtggcttggtacactgcttgggctgtgtagtcctgcccacctctgctcattttctctggatagagttttaagatcgcagctagtttcccctgccatggcaggtatgactatggggctggagcatccaggagagggggatgcttgccctcttggagtccctgtactaaatgtaacgtcctcccttgctccagcagggctccattctgacctggagtccaccagcaccatgcaggaggactggaggggaggaaggctgctttaggcagtccaaggtgctccctccccctctagcaatggcaaggtcttcccagctcctgtcaggactgtcttcaaaggccatctctgcccaccgcatatgtgacgaagctagcctttcccaggagcctagtgccgcctcagctgggcctggtgctcccgtgcctggggttagtgtgagggctcaggaggaggagttcccaagggactgagtagaggcttccaggtggctgaccagtactgacctgacacggtccctgatggactcctcatggggcgggcatcgggtggccttctctagctttccttccagccccatctgtaggcttagtcttaccatagtgtgagggcaccaggcgagttagcccccaatatcttttggggaggggtgtctttgggactgtgccaatctggctattcatccgtctctaccccaagtgcggcttctagggtcacctctgaggagagctgctgggatgctggggtagctgaggggaggttgtggggagtttgcagtgcatctaggaactagctcactgcaggtggggctgaagctggattcagtcatgggaggttccctgtgggctttcctttccaaagactaggcttgttttccctagaaactaaggtgctggggactgggtgacagccacctctcagcaggtgccgtgtaagtgtgaagatttgtgaagatctgtggcccttgcccaggggaggtagctggtgctagcaccagggtcacagaactaaggcctcctgaggaggcacacataccagcggggtcccaagctggtgctaattcctctcataggacccagtaagcccgggagcctgcagtcggctgtttctctgctgtggaataccctgttagaaagctgaggtcccctaaagagcctcctggaccgagtcacttttgtggccgggctgagtatggcagcaggtagaaagatccgcaggcataaagatccctgtggcttttggcaaatgactgaagtgtggatgcaggagcaatgagtggaggccaacatcaacagcatctaccctccccaatagtgtctctgttctgggggtgctgtggtcctcacttgctggcttaggattggagttaggctgagccactttgaaggagtgtgtttacaggaagatacaaattgttggtttctctattctttactgtacagtgtgtccagagagtagaagaaaattcctgggcctgtctggttggtgcagagcttttatggcactacaaggtcttcaggaactgtcactgtaagagccctggctgtgagctggggctacgtgaccactgcagctgagtttccaagcaggcctgtgtttggatctgagtttgacgcttggcggctcccagggcttgggatgagtggcagtgctgacttgttcacttggcagagccaccctgtgaggaggtggacttagtggccctttccaattgatcctgtcccatccaggcagtatcagcactaggagcagtgctgggtcagaagtccgaaggaaaggactcggttgatcggggtcattgtgagcttgtgtatgtttgtgtagagaaagctgaactttgggaggaaaggccttgtccttgcaggcttccaagacaccgctaaggttagcctttggttggagacaagtctctagcccagacactggagtaggcgctgagaccaagaggcaggccaagatggagatctatggaaggccctttgctggtagggattcaaagcttctccaggggaccccagaactctattaactggctcccctaccgggacaagcttcgtttgtgggctcatagagtggccatagagtgggtttaacccagcaccaagcctgagggcatgagcagtggtctaaagtctcacatagtcccattttacagactaatttgattgcccggccgtagaattctacagaaacaacaatcactgaaattccttagaatacgcagagggaggaggtgactcaacacggtgctaaacacattttattaaaaatatatattgttaaattaagtctgctgtctggacaatgatgttctgttttgttttcttgtagtggagtttaaaagagactattattttactctgatatattattattaaaaaggcattttaactttgtacttgaaaactaagtgagcgatttcacgtgttttagctgagacatcgaagtagcgggtgcttacttgtgggaaatcatgtattcatactacaacataaactccgtagctgatttggtaataacttttactgttacagacgaattcgctttgaccccggtggcagagcgctttgactccacattccacgcagctaagtgcaggaccttccagaaccttccccggtcccccctggtcctctttcggttcctcttcctagctctctcttttcctaattcttttttttaagaattgtcgtttgtttttcctggtcccggcttccttctaccactgtattttttgttagggttgcttctctatttattgacagtaataatgtacatttcacagtctggttctgggacagccaggaggggtgtggggggctgcatacagcttgctgcactctccggtgtcctgttgtatctctgtgtacgtgatgcttgtgacatagctgttgaagaaatattaaaaggtcaatgcgtacagcaggtgtagagagtccgtcagttccgcttcatcactttttttttttttttttgtgcccagaagaatataataaagctcctttctaatgtacttgtgctggagaacacttgaataaatggactgtttttgtgcaaaaagaaaaacggaaaaaaaaaaaaaagcaccgtcataatgtccttttgtcctgcgactcctttcccctgatgtggccactcacagtcctccagtgggctgaagtgggtgcagtttacgttgggagggtctgggaaatggatttctaaagtatctctcatatgggtgttaactaggggttgggacccaagggcagagggagtttggttcaagatcaaaaacaggttagactaaggtgaagttgtctcgtttacttcccaaaccagcagattctcgagaccagagcaggcaacagactggcatcctggttagctggcagtaggaccagcagggacttatggatgccagagtccagaaggaagaagtcagcaaaggagagaggcagctggggctgggggaatcctggaagaaccttgcgtttaaaggagatatttgcaaacacttgacaggtgacccaagggctgggatcagtggccaatagggctactgctggtctgagcataggctgcctggcagggccttgcagcctgaggagttgtccaccgtgtctctgccatacctatctagaagggccgtctgtatgcggggtctgtctgtcctgcatatctccgggagtagccagcaggcttggctggggttccactcctaaggactgtccacccgtttcttccagcaaactttctggacggttggatagggagagaccgtgtggcggtccttgagcagactgggaaagtttggctaaacaggttttctcatgggcactttttcttgctttttctttgacaaggttgtaaatgtgtatttggcttttactctactttttttcttgatgtttttcaatgttgatgtggaatcttactttcaaatggctgcatggcattttcttgttgaatgtttgtttatatatattttattttcgctataaatagagcttcaataaacatctttatgttttggcttccc

Website prediction Results：http://www.mirdb.org/cgi-bin/search.cgi


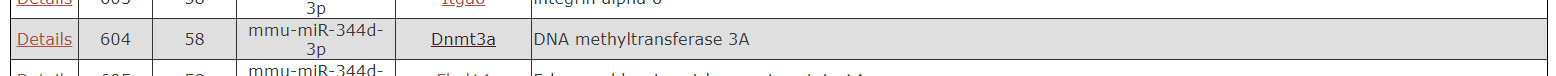


https://www.ncbi.nlm.nih.gov/gene?cmd=Retrieve&dopt=full_report&list_uids=13435


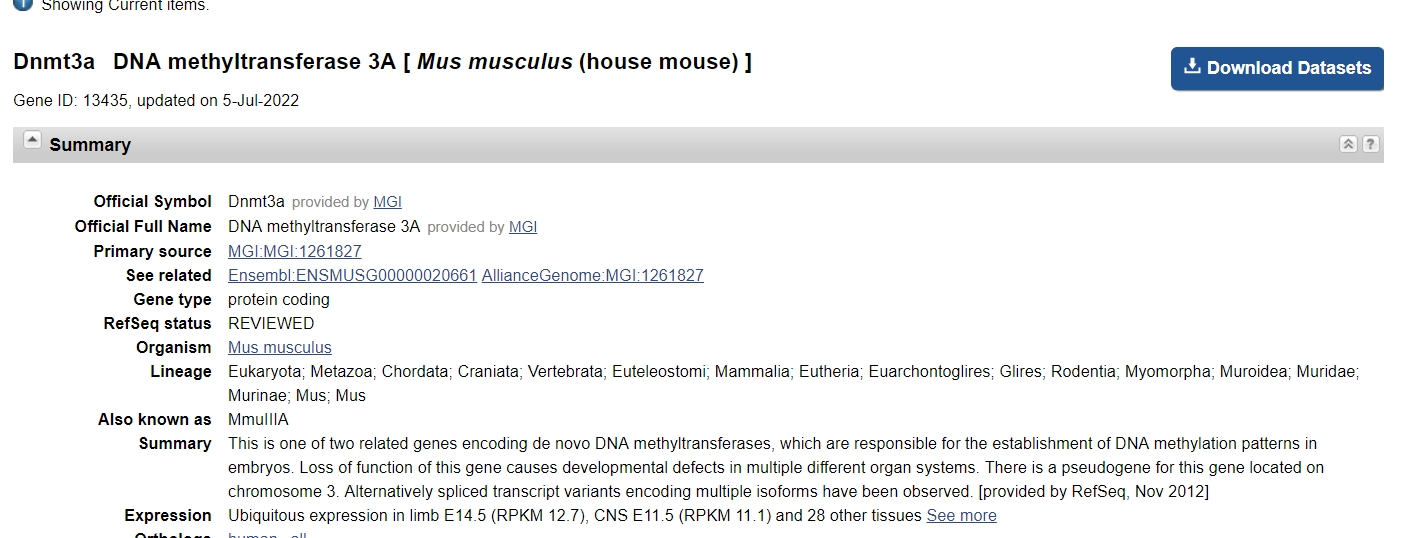

Supplement: Figure S5 — Raw data: Figure 5 A-B [file peerj-11-14838-s022.zip › Figure 6/A/mmu-miR-344d-3p Raw data for binding site prediction.docx]
